# O-GlcNAcylation protein disruption by Thiamet G promotes changes on the GBM U87-MG cells secretome molecular signature

**DOI:** 10.1186/s12014-021-09317-x

**Published:** 2021-04-26

**Authors:** Maria Cecilia Oliveira-Nunes, Glaucia Julião, Aline Menezes, Fernanda Mariath, John A. Hanover, Joseph Albert Medeiros Evaristo, Fábio César Sousa Nogueira, Wagner Barbosa Dias, Denise de Abreu Pereira, Katia Carneiro

**Affiliations:** 1 Laboratory of Cell Proliferation and Differentiation, Institute of Biomedical Sciences, Federal University of Rio de Janeiro, Rio de Janeiro, RJ Brazil; 2 Laboratory of Cell Biochemistry and Molecular Biology, NIDDK, NIH, Bethesda, MD USA; 3 Laboratory of Proteomics/LADETEC, Federal University of Rio de Janeiro, Rio de Janeiro, RJ Brazil; 4 Laboratory of Structural and Functional Glycobiology, Institute of Biophysics Carlos Chagas Filho, Federal University of Rio de Janeiro, Rio de Janeiro, RJ Brazil; 5 Program of Cellular and Molecular Oncobiology, Membrane Receptors and Cancer Group, Research Coordination, National Institute of Cancer, Rio de Janeiro, RJ Brazil; 6 Postgraduate Program in Medicine (Pathological Anatomy), Federal University of Rio de Janeiro, Rio de Janeiro, RJ Brazil; 7 Present Address: Immunology, Microenvironment and Metastasis Program, The Wistar Institute, Philadelphia, PA USA

**Keywords:** Glioblastoma, Secretome, O-GlcNAcylation, O-GlcNAcase

## Abstract

Glioblastoma (GBM) is a grade IV glioma highly aggressive and refractory to the therapeutic approaches currently in use. O-GlcNAcylation plays a key role for tumor aggressiveness and progression in different types of cancer; however, experimental evidence of its involvement in GBM are still lacking. Here, we show that O-GlcNAcylation plays a critical role in maintaining the composition of the GBM secretome, whereas inhibition of OGA activity disrupts the intercellular signaling via microvesicles. Using a label-free quantitative proteomics methodology, we identified 51 proteins in the GBM secretome whose abundance was significantly altered by activity inhibition of O-GlcNAcase (iOGA). Among these proteins, we observed that proteins related to proteasome activity and to regulation of immune response in the tumor microenvironment were consistently downregulated in GBM cells upon iOGA. While the proteins IGFBP3, IL-6 and HSPA5 were downregulated in GBM iOGA cells, the protein SQSTM1/p62 was exclusively found in GBM cells under iOGA. These findings were in line with literature evidence on the role of p62/IL-6 signaling axis in suppressing tumor aggressiveness and our experimental evidence showing a decrease in radioresistance potential of these cells. Taken together, our findings provide evidence that OGA activity may regulate the p62 and IL-6 abundance in the GBM secretome. We propose that the assessment of tumor status from the main proteins present in its secretome may contribute to the advancement of diagnostic, prognostic and even therapeutic tools to approach this relevant malignancy.

**Supplementary Information:**

The online version contains supplementary material available at 10.1186/s12014-021-09317-x.

## Introduction

Glioblastoma (GBM) is a grade IV glioma highly aggressive and refractory to the therapeutic approaches currently in use in the clinic [27]. The poor prognosis is due in large part to tumor heterogeneity observed at different levels, such as phenotypic, transcriptional, epigenetic and metabolic [46]. Altered sugar metabolism is a hallmark in tumors, which in general produce high levels of lactic acid through aerobic glycolysis. Besides glycolysis, about 2 to 5% of the total glucose transported into the cell enter the hexosamine biosynthetic pathway (HBP) [30]. The limiting enzyme of HBP is glutamine: fructose-6-phosphate amidotransferase (GFAT), which converts fructose-6-phosphate into glucosamine-6-phosphate. The final product of HBP is the UDP-*N*-acetylglucosamine (UDP-GlcNAc).

UDP-GlcNAc is the monosaccharide donor of O-GlcNAcylation and its biosynthesis involves the metabolism of glucose, amino acids, fatty acids and nucleotides, being considered an excellent sensor of the nutritional status of the cell [12, 18]. When this glycan is covalently beta-linked via N-acetylglucosamine to the polypeptide by the hydroxyl group of a serine or threonine residue, it is called O-GlcNAc. Protein O-GlcNAcylation is regulated by the action of two antagonistic enzymes, O-GlcNAc transferase (OGT) and O-GlcNAcase (OGA), and different reports in the literature have shown that the O-GlcNAcylation is related to different types of cancer [1, 11, 21, 39, 42]. In fact, any disruption in protein O-GlcNAcylation levels, as well as in the activity of OGT and/or OGA, have been implicated in metastasis and cell transformation processes in different types of tumors [10, 18, 28]. However, few studies have been dedicated to better understand the role of O-GlcNAcylation in GBM.

Protein O-GlcNAcylation is an important post-translational modification (PTM) involved in different aspects of cell signaling, being related to mechanisms of cell proliferation and differentiation, being as ubiquitous as phosphorylation [5, 20]. In addition, O-GlcNAcylation and phosphorylation can compete by the same amino acid residue, or at close sites, considerably increasing the complexity and functional diversity of a given signaling pathway [9].

A pioneering study in adipocytes demonstrated that modulation of O-GlcNAc was able to alter the secretome, indicating a possible role of this PMT in cellular secretion [50]. The secretion of extracellular mediators by the tumor is an important strategy to modulate the behavior of host cells, to promote angiogenesis, to suppress the immune response and to modify structural aspects of the tumor microenvironment, such as the extracellular matrix [2]. Among the molecules secreted by gliomas, there are proteins, nucleic acids, lipids and metabolites, which promote several alterations that support tumor evasion and, consequently, may emerge as putative targets for therapeutic approaches [49]. Thus, mapping the protein repertoire found in tumor secretions, using proteomic tools, may emerge as an important experimental approach in understanding the tumor response to different disruptions in the intracellular microenvironment. Another important advantage is the development of less invasive diagnostic and/or prognostic strategies since the mediators secreted by the tumor can be collected from distal body fluids, such as cerebrospinal fluid.

Thus, in the present work, we sought to characterize the proteins present in the secretome of GBM upon protein O-GlcNAcylation disruption using a label-free proteomics strategy. For this, we used Thiamet G (TMG) for the pharmacological inhibition of OGA activity (iOGA) and hence promotion of protein hyper O-GlcNAcylation. Then we characterized the GBM secretome by label-free liquid chromatography-tandem mass spectrometry (LC-MS/MS) and analyzed the protein repertoire using bioinformatics tools. *In silico* analyses of the mass spectrometry data showed that the GBM secretome mainly contained proteins related to classical cell signaling pathways, such as angiogenesis/vasculogenesis, cell migration and signaling via p53. After inhibiting OGA activity, we observed qualitative and quantitative changes in the abundance of 51 proteins, showing that OGA activity is necessary to maintain the molecular signature of the GBM secretome. We also observed that after the inhibition of OGA activity, the U87-MG GBM cell line increased the p62 levels and exhibited reduced radioresistance. These findings suggest that mapping of molecules secreted by the tumor in peripheral body fluids can emerge as an important strategy to better understand tumor behavior and thus improve the therapeutic strategies currently used in the clinic for GBM.

## Material and methods

### Cell line

The GBM cell line U87-MG was obtained from the Rio de Janeiro Cell Bank and genotyped using microsatellite markers of human lineages at the Macromolecular Metabolism Laboratory Firmino Torres de Castro (Carlos Chagas Filho Biophysics Institute, Federal University of Rio de Janeiro).

### Cell culture and drug treatment

The cells were platted at a density of 7 × 10^5^/mL in Dulbecco's Modified Eagle's Medium (DMEM; Sigma-Aldrich), supplemented with 10% Fetal Bovine Serum (FBS), (LGC Biotecnologia) and 0.1 mg/mL penicillin/streptomycin (PS), in 150 cm^2^ flasks (Corning), and incubated at 5% CO_2_ and 37 ºC until confluence of 90%. The flasks were then washed with phosphate-buffered saline (PBS) 1% plus 2 M NaCl twice and with 1% PBS in order to remove the excess FBS and salts in the culture medium. The culture medium was replaced by DMEM without phenol red and FBS, and supplemented with 1 μM TMG (Sigma-Aldrich; iOGA experimental group) or dimethyl sulfoxide (DMSO; control group). For each condition, two flasks were generated and the conditioned medium (CM) was collected separately after 72 h of treatment at 5% CO_2_ and 37 ºC. Each biological replicate was created from flasks of independent cultures generating a total of 2 biological replicates (DMSO1, DMSO2; iOGA1 and iOGA2). Due to the fact that GBM displays a range of different phenotypic, genetic and epigenetic subtypes, also represented by different GBM cell lines, we chose to use only one GBM cell line (U87-MG) to better explore a putative role for OGA activity without aiming to compare, at this first moment, this putative role among different GBM subtypes.

### Immunocytochemistry staining of adherent cells on coverslips

Cells were plated on coverslips at 2.5 × 10^4^ and treated accordingly for 72 h. Then the cells were fixed in 4% paraformaldehyde/PBS for 5 min at room temperature (RT), washed three times for 5 min each with PBS, permeabilized in PBS/Triton X 0,3% for 3 min and blocked in PBS 5% BSA for one hour. After blocking, the coverslips were washed with PBS three times, at every 10 min, followed by primary antibody incubation. Immunostaining was performed using primary anti-O-GlcNAcylation (clone RL2, mouse, sc-59624 Santa Cruz) at 1:200, for two hours at RT. The coverslips were then washed again three times with PBS, at every 10 min, then incubated with secondary antibody conjugated to Alexa-633 fluorochrome (Molecular Probes). After another round of three PBS washes, cells were incubated with DAPI for 10 min at RT and the slides were mounted in ProLong (Invitrogen) mounting media.

Images were acquired with Leica TCS SP5 AOBS and the images were analyzed using ImageJ software (1.51 K Java 1.8.0_66 64-bit version).

### Protein sample preparation

The obtained CM was stored at − 80 °C after collection, in order to properly disrupt the extracellular vesicles. Approximately 40 mL of the CM from each group was centrifuged at 1200 rpm for 10 min at 4 °C. Then, the volume was concentrated to 250 µL in a 15 mL AMICON 3 KDa tube (UFC900324, Millipore) by centrifugation at 5000 rpm for 30 min. Halt 1 × protease inhibitor (Halt Protease Inhibitor Cocktail, Thermo Fisher) was added to the concentrated CM and protein quantification was performed using Qubit Protein Assay Kit (Q33211, Thermo Scientific) according to the manufacturer's instructions.

### Protein hydrolysis and liquid chromatography-tandem mass spectrometry

After protein quantification, the protein digestion in solution protocol using trypsin was followed 100 µg of protein were used in 20 µl of 7 M Urea and 2 M Thiourea in 0.2 M in HEPES. Then we added dithiothreitol (DTT) at a final concentration of 10 mM and the solution was incubated for one hour at 30 °C in the Thermomixer (Eppendorf) without stirring. After incubation, iodoacetomide solution (IAA) was added at a final concentration of 40 mM and the incubation continued for 30 min at room temperature, in the dark. We then diluted the obtained solution at 10 times ratio with standard LC–MS deionized water and added the trypsin solution (Sequencing Grade Modified Trypsin, PROMEGA V511A) at the final concentration of 1:50 trypsin/protein (w/w). Addition of trypsin was followed by overnight incubation at 37 °C in the Thermomixer, under 900 rpm rotation.

After incubation, the samples were resuspended in 1% formic acid and passed through C18 microcolumns (ZipTip) for peptides desalting. The samples were dried using speed vac and frozen at −  20 ºC. Next, the peptides were solubilized in 0.1% formic acid and analyzed using an Easy1000 nanoLC system (Thermo Scientific) coupled to an Orbitrap Quadrupole (Q Exavtive Plus, Thermo Scientific). For each sample, a volume of 9 μL (1 μg of peptides) was applied to a trap column with 200 µm of internal diameter and 2 cm long packed with Reprosil-Pur C18 resin (Dr. Maisch), with 200 Å pores and 5 µm granulometry (packaged in the laboratory). The peptides were eluted in an analytical column with 75 µm in diameter and 25 cm in length packed with Reprosil-Gold C18 resin (Dr. Maisch), with pores of 300 Å particle size of 3 µm. Peptide separation was performed using a gradient from 95% solvent A (ACN 5% and formic acid 0.1%) to 20% of solvent B (ACN 95% and 0.1 formic acid) for 120 min, 20–40% solvent B in 40 min and 40–95% solvent B in 7 min. After separation, the column was equilibrated with solvent A. Xcalibur software (version 2.2) was programmed to operate in data-dependent acquisition (DDA) mode.

The spectrum of MS^1^ was acquired with a resolution of 70,000 at m/z 200. The reading of the MS^2^ spectrum comprised ions with a range of m/z 375 to 2000. The 15 most intense ions were fragmented and then submitted to MS^2^ acquisition, using induced collision dissociation (HCD) and 200–2,000 m/z. MS^2^ resolution was 17,500 at m/z 200, automatic gain control (AGC) of 10^5^ ions, maximum injection time (IT) of 100 ms, 2 m/z ion isolation window, normalized collision energy (NCE) of 30, dynamic exclusion time was 45 s. Peptides with charge + 1 and undetermined were rejected. Protein quantification was done in the Proteome Discoverer 2.1 using the Precursor Ions Area Detector node, where the average of the 3 most intense peptides was used for protein quantification, being considered only unique peptides. Normalization of quantitative data was done using Perseus v.1.6.10.50, the data were transformed into log2, normalized by subtracting the median. For each biological sample, three experimental replicates (three injections) were made. To check for reproducibility between control or iOGA biological replicates, we performed the Principal Component Analysis (PCA) and Venn Diagram analyses. These careful analyses showed that control replicates were reproducible as well as iOGA treated replicates (Additional file 1). In addition, we chose to use only the 808 common proteins found in the Venn diagram between control replicates 1 and 2 and the 775 common proteins found between iOGA (data not shown).

### Data analysis

Raw files were processed by the Proteome Discoverer (PD) software (version 2.1; Thermo Scientific) and spectral data were searched using Sequest HT-Percolator Validator algorithm. The UniProt database limited to Homo sapiens reference proteome set was downloaded from Uniprot consortium in June 2017. The parameters used in PD Sequest HT node were: full-tryptic search space, up to two missed cleavages allowed for trypsin, precursor mass tolerance of 10 ppm, and fragment mass tolerance of 0.05 Da. Carbamidomethylation of cysteine was included as fixed modification, and methionine oxidation and protein N-terminal acetylation were included as dynamic modifications. To estimate the false discovery rate (FDR) of < 1% and protein inference we used the node Percolator present in the PD software using maximum parsimony.

### In silico analysis

To characterize the biological process and signaling pathways we have used PANTHER GO (http://pantherdb.org) and The Human Reference Interactome (http://www.interactome-atlas.org/) repositories. The UniProt database was used to investigate signal peptides, and the Vesiclepedia (http://microvesicles.org) to search for proteins found in microvesicles. The found proteins were identified using PD software (Thermo Fischer). To analyze the statistical significance of protein quantifications in volcano plot and heatmap, we used Perseus software (version 1.6.10.50). Protein distributions were done with the platform InteractVenn diagram (http://www.interactivenn.net/citation.html).

### Cell viability and cell death assay

For flow cytometry assays, 5 × 10^5^ cells were platted in six-well plates and treated for iOGA or with DMSO for 72 h. After treatment, the cells were trypsinized, centrifuged, washed with PBS and counted in the Neubauer chamber with trypan blue. Apoptosis was checked using annexin-V FITC (BD Biosciences) detection kit, according to the manufacturer's instructions. Apoptotic and/or necrotic cells were detected under annexin V-FITC and propidium iodide (PI). The analyses were performed by flow cytometry in a FACSCanto device, using FACSDiva software (version 8.0.1; BD Biosciences). A total of 30,000 events were collected for each sample. The outcomes were expressed as an average of the percentage of cells distributed in the different quadrants, which were later normalized by the total number of cells obtained by counting in a Neubauer chamber.

### DNA content analysis

Cell cycle analyzes were performed indirectly, from the quantification of DNA content by flow cytometry. For analysis, 10^6^ U87-MG cells were resuspended in 400 µl of Vindelov buffer and incubated for 15 min at 4 ºC, protected from light. The samples were acquired in the FACSCanto device (BD Biosciences), using FACSDiva software (BD Biosciences). A total of 20,000 events were collected for each sample. Eventual cell clusters were removed from the analysis using the cell scattering profile (using parameters FSC-A and FSC-H). The analyses were expressed as an average percentage of cells distributed in the different quadrants.

### Western Blotting experiments

5 × 10^5^ cells/well U87-MG cells were platted in six-well plates and treated with TMG or DMSO for 72 h. After incubation, cells were trypsinized, washed with PBS twice and the pellets were stored at − 80 °C until protein extraction with RIPA buffer supplemented with Halt phosphatase inhibitor (Thermo Scientific) and protease inhibitor cocktail (cOmplete, Roche). Proteins were resolved by 4–10% SDS-PAGE gels and transferred to Nitrocellulose membranes (Millipore EMD) at 100 V, on ice, for one hour. The membranes were blocked for 1 h at room temperature, with 5% milk (for antibodies not detecting O-GlcNAcylation), or with 3% BSA (for antibodies detecting O-GlcNAcylation). The primary antibodies were incubated in PBS with 0.1% Tween (PBT) at 4ºC, overnight. The antibodies used were: anti-OGT clone EPR12713, anti-rabbit, 1: 1000 (ab177941, Abcam); anti-OGA, clone EPR7154 (B) anti-rabbit, 1: 1000 (ab124807, Abcam); anti-O-GlcNAc RL2, anti-mouse, 1: 1000 (MA1-072, Thermo); anti-α-tubulin, DM1α clone, anti-mouse, 1: 2000 (T9026, Sigma); and anti-p62/SQSTM1, anti-rabbit, 1: 2000, (PM045, Medical and Biological Laboratories Co). The membranes were kept under low agitation. The incubation was then carried out with the respective secondary antibodies (IR-Dye^®^ 680RD Goat anti-rabbit, LI-COR, 1: 10,000; IR-Dye^®^ 800CW Donkey anti-mouse, 1: 10,000), at RT for two hours, under gentle shaking. The chemiluminescence was developed using SuperSignal West Pico (Thermo Scientific) and the images were obtained in an Image Odyssey CLx LI-COR device. The images were acquired, analyzed and quantified using the Image Studio LI-COR software.

### Ionizing irradiation and MTT assay

U87-MG cells were platted at 1.3 × 10^4^ cells/well in a 96-well plate and treated with TMG or DMSO for 72 h. The cells were irradiated in a single dose of 10 Gy, with a 6MV linear accelerator in a field equivalent to 25 × 25 cm^2^ and adjusted to the window size and the surface distance of 70 cm. After irradiation, a 3-(4,5-Dimethylthiazol-2-yl)-2,5-diphenyltetrazolium bromidefor (MTT) assay (Sigma-Aldrich) was performed to check cell viability, according to the manufacturer's protocol, at times 0, 24, 48 and 72 h after irradiation. The absorbance was read in a plate reader at 540 nm. To quantify the decay in the number of viable cells after irradiation, the numbers obtained by counting in a Neubauer chamber were used, at the times of 24, 48 and 72 h after irradiation.

## Statistical analysis

Statistical analysis was performed using the GraphPad Prism software (version 8.4.2), using ANOVA with Bonferroni's post-test. Western Blots were analyzed by unpaired t tests. Differences were considered significant when p < 0.05.

Raw data: submission details: https://www.ebi.ac.uk/pride/login; Username/email: reviewer16105@ebi.ac.uk; Password: YPJl97M5 Project Name: O-GlcNAcase activity is necessary for the molecular signature of the Glioblastoma secretome; Project accession: PXD019496; Project DOI: Not applicable.

## Results

### Inhibition of OGA activity increases the number of viable GBM cells

**Fig. 1:**
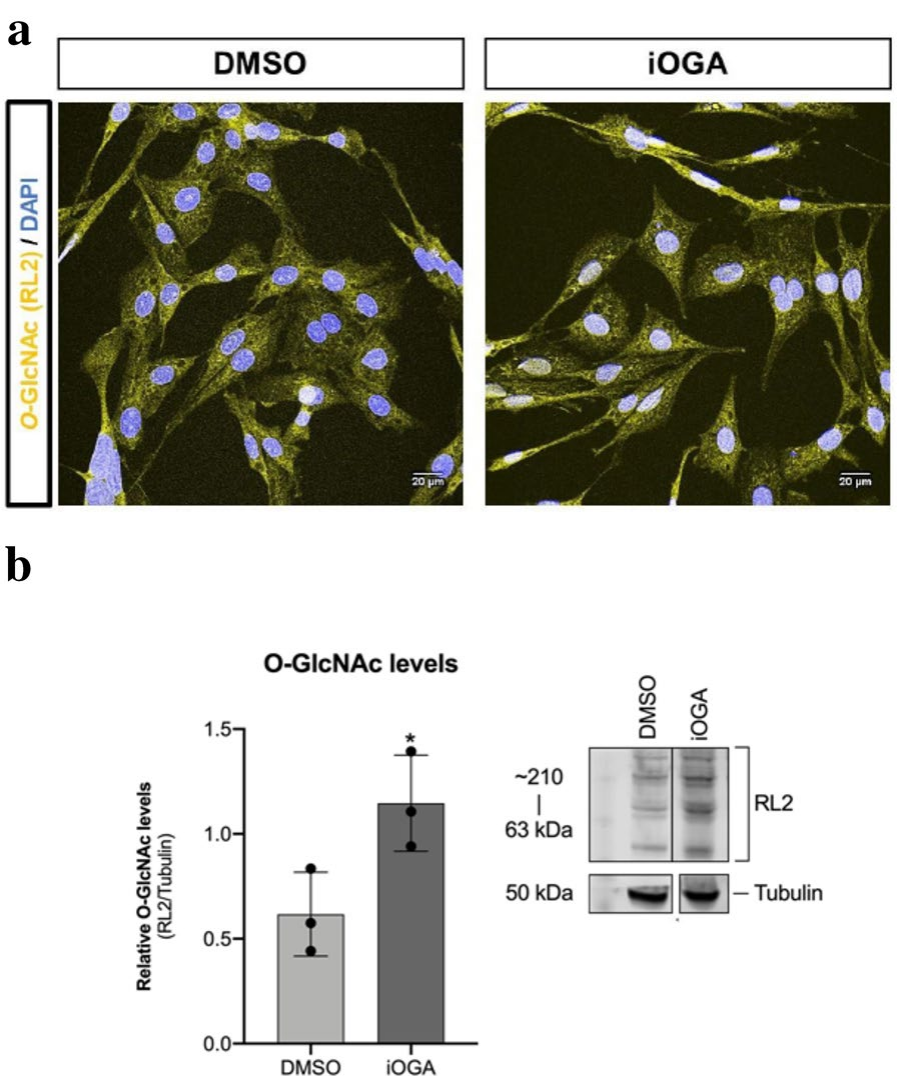
OGA inhibition increases protein O-GlcNAcylation without morphological changes in GBM cell line. **a** Confocal fluorescence microscopy of control and iOGA U87-MG cell upon 72 h showing cytoplasmic and nuclear O-GlcNAc staining (yellow); cell nuclei (blue) **b** Western Blotting and quantification of the protein O-GlcNAcylation. N = 3; * *p* < 0.05

**Fig. 2:**
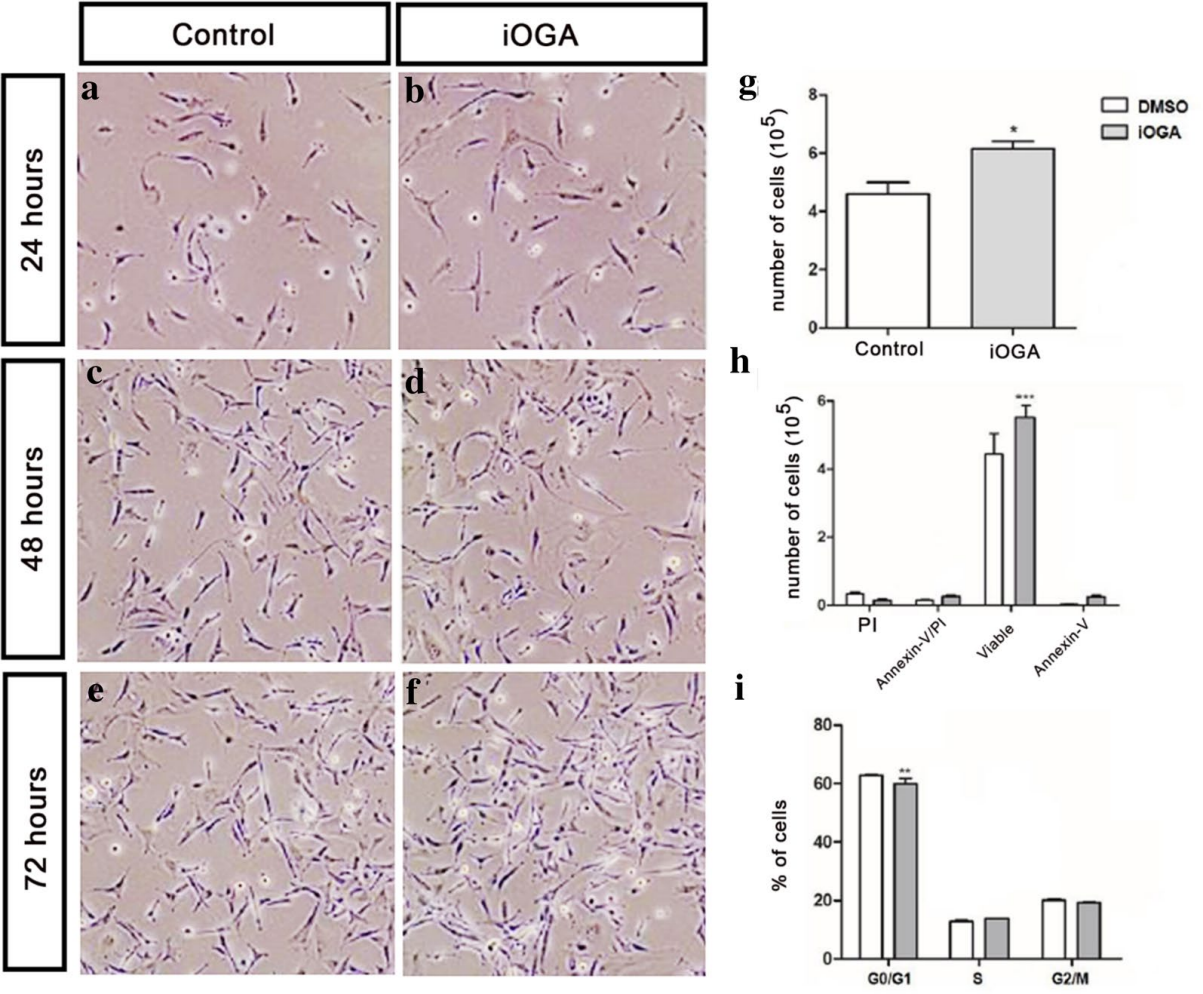
The inhibition of OGA activity increases the number of viable GBM cells in vitro. **a–f** Optical microscopy in bright field showing cells of the control group and iOGA group during 24 h, 48 h and 72 h of treatment. **g** Counting of viable cells in Neubauer chamber by trypan blue exclusion after 72 h of treatment; **h** Quantification of cell viability assay by flow cytometry after 72 h of treatment; **i** Quantification of cell cycle assay by flow cytometry after 72 h of treatment. N = 3; * *p* < 0,05; ** p < 0,01; *** p < 0,001

Although O-GlcNAcylation has been shown in different types of tumors, few studies have evaluated the role of this PTM in GBM. Treatment of GBM cells with TMG to induce iOGA resulted on no apparent morphological changes (Fig. 1a), but on a two-fold increase of the O-GlcNAc levels with (Fig. 1b). In addition, upon 72 h of culture (Fig. 2a–f), we observed a significant increase in the number of viable cells in the iOGA group (Fig. 2g, h). The iOGA cells also showed a slight decrease in the total number of cells in the G0/G1 phase of the cell cycle (Fig. 2i).

### Inhibition of OGA activity changes the GBM secretome quantitatively and qualitatively

**Fig. 3:**
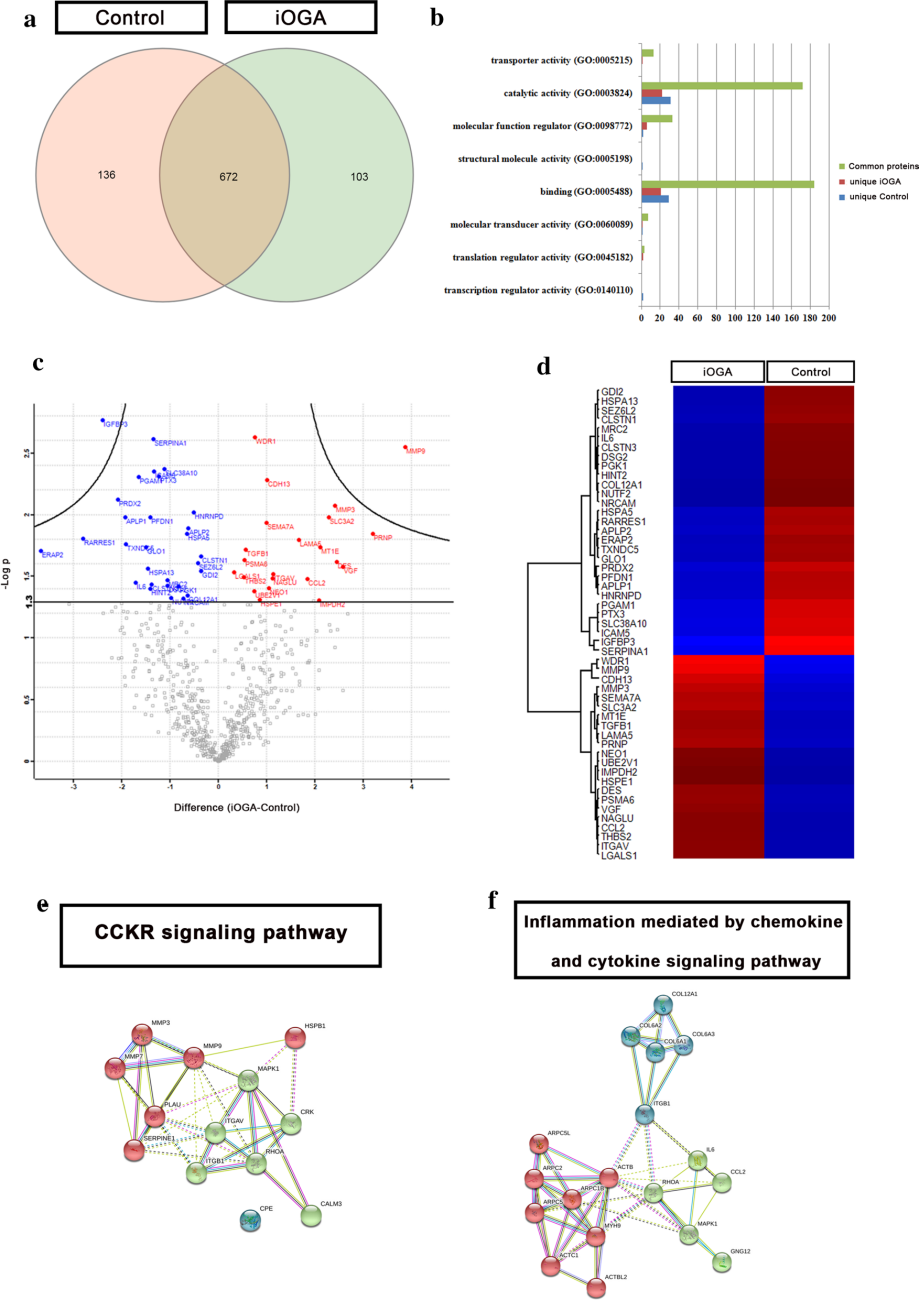
The inhibition of OGA activity alters the molecular signature of GBM secretome. **a** Venn diagram showing the distribution of proteins identified in the label free proteomic quantifications between control and iOGA groups. **b** PANTHER GO molecular function classification of the proteins identified and previously distributed between the control (blue) and iOGA (red) groups. **c** Volcano plot showing the 51 proteins whose abundance has changed significantly upon iOGA treatment. *p* < 0.05 (**d**) Heat map grouping the 51 proteins with altered abundance according to up regulation (red) or down regulation (blue) between control and iOGA groups. **e** String illustrating the interactions detected between proteins that belong to CCKR signaling pathway and (**f**) Inflammation mediated by chemokine and cytokine signaling pathway

Although it is well established that O-GlcNAcylation is highly responsive to nutrient levels and microenvironment, it is unknown whether modulation of O-GlcNAc levels have an impact on the GBM secretome. Label-free LC-MS/MS quantitative proteomic analysis of control and iOGA groups allowed identifying a total of 911 proteins, of which 136 were unique to the control group, 103 were unique to the iOGA group and 672 proteins were common to both groups (Fig. 3a ; Additional file 2). While most proteins showed binding or catalytic activity (Fig. 3b), we observed that 206 proteins participate in 104 distinct signaling pathways identified by the PANTHER GO classification system. Out of this pool, 19 proteins were unique to the control group, 36 were unique to the group iOGA and 156 proteins were common to both groups (Additional files 3, 4, 5). Among the signaling pathways identified and which have been already documented to correlate with GBM biology, we highlight the angiogenesis, cadherin signaling pathway, epidermal growth factor (EGF) receptor signaling pathway, hypoxia response via HIF activation, Notch signaling pathway, PI3 kinase pathway, vascular endothelial growth factor (VEGF) signaling pathway, Wnt signaling pathway and p53 pathway (Additional file 6).

We next analyzed the proteins in common to both groups to check whether there were statistically significant differences in protein abundance between the control and the iOGA group. The analysis of the volcano plot revealed that 51 shared proteins showed significant fold-change (Fig. 3c; Additional file 7) and the heat map grouped these proteins according to up or downregulation within each group (Fig. 3d).

At this point, we analyzed the signaling pathways again to understand whether the quantitative changes observed could putatively disrupt the signaling pathways to which they belong to. The signaling pathways that presented the higher number of proteins up or downregulated were the cholecystokinin (CCKR) pathway (Fig. 3e; MMP9, MMP3 and ITGAV, being all up regulated) and inflammation mediated by chemokine and cytokine signaling pathway (Fig. 3f; COL12A1, IL6, CCL2). Details on fold-change can be found on Additional file 7. Noteworthy, the mitogen-activated protein kinase 1 (MAPK1) was absent in both signaling pathways under iOGA and an integrin-alpha 2 protein was detected in the inflammation mediated by chemokine and cytokine signaling pathway under iOGA (Additional file 7). Then we analyzed the proteins that were not identified as participating in any signaling pathway. Among the upregulated proteins, 14 of them were not found to be part of any signaling pathway, while 24 of the downregulated proteins were not part of any signaling pathway (Additional file 7).

Thus, our analysis suggests that significant differences in the abundance of proteins that participate in the same signaling pathway can disrupt its pattern of activation upon OGA inhibition.

### Increase of O-GlcNAcylation modulates secretome proteins in GBM

As we are studying extracellular proteins, we considered the classification of proteins according to their secretion route. For proteins secreted by the classic secretion pathway, we considered the presence of signal peptide for the classical endoplasmic reticulum (ER)-Golgi pathway [35], by searching by keywords at UniProt database. As for proteins that lack a signal peptide, we considered the presence of the protein in microvesicles according to the database Vesiclepedia.

**Fig. 4:**
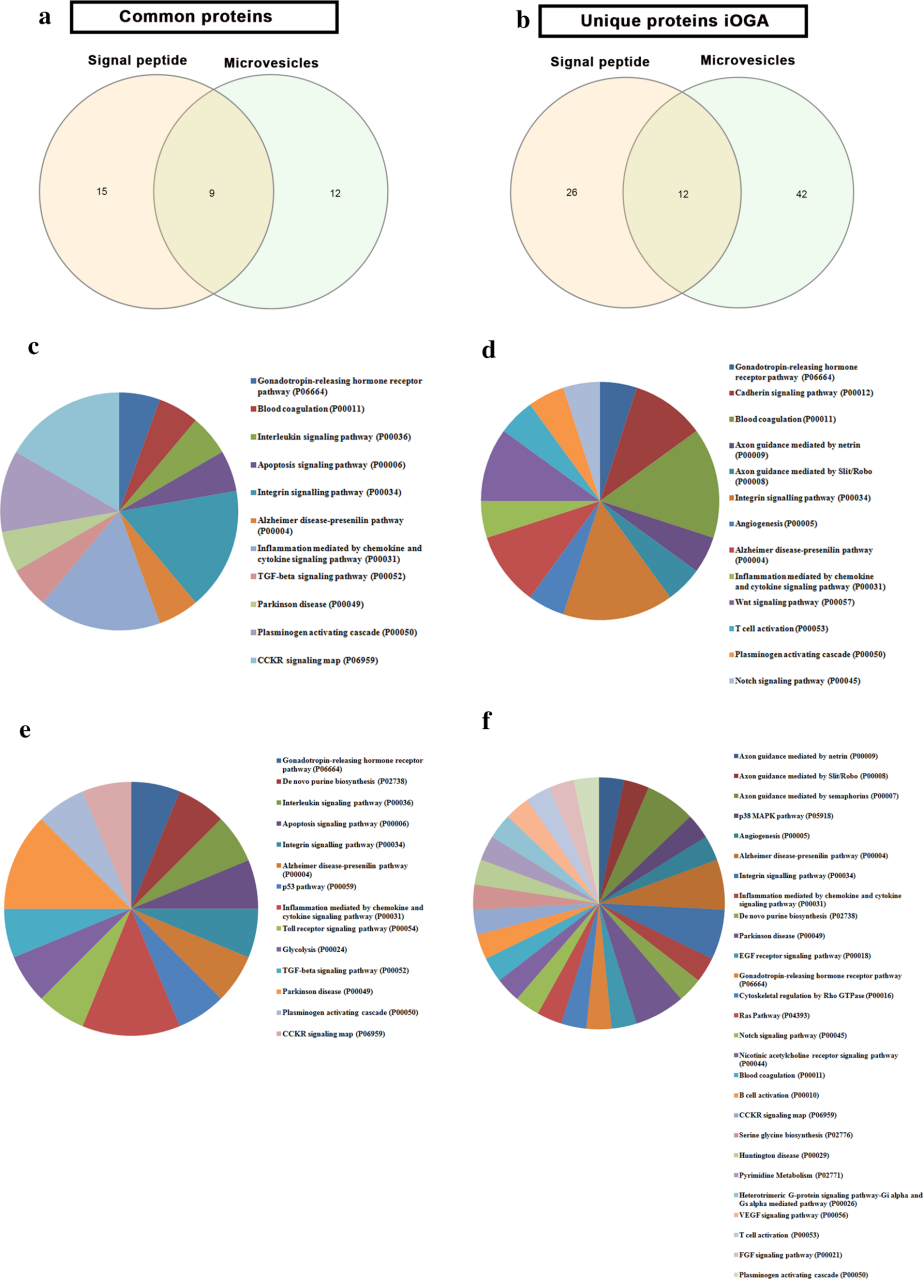
OGA inhibition alters protein distribution in GBM microvesicles. In silico analysis showing the number of proteins displaying signal peptide or detected in GBM microvesicles in control (**a**) or iOGA (**b**) group. Distribution of proteins displaying signal peptide in different signaling pathways in the control (**c**) or iOGA (**d**) group. Distribution of proteins found in GBM microvesicles in different signaling pathways in control (**e**) and iOGA (**f**) group

Of the 51 shared proteins between the two groups, we observed that 47% (24/51) had signal peptide, 41% (21/51) had already been reported to be present in GBM microvesicles and 17% (9/51) had both characteristics. In the proteins exclusive to the iOGA group, we observed that 40% (42/103) had signal peptide, 51% (53/103) had already been reported in GBM microvesicles and 11% (12/103) had both characteristics (Fig. 4a, b). Among the 51 proteins harboring a signal peptide, 24 proteins were distributed in 11 signaling pathways (Fig. 4c), whereas in the 38 proteins exclusive to the iOGA group were distributed in 13 signaling pathways (Fig. 4d). After a cross analysis between the proteins that have already been identified in GBM microvesicles and the proteins found in our secretome analysis, we observed that of the 21/51 proteins were distributed in 14 signaling pathways (Fig. 4e). Furthermore, 54/103 of the proteins unique to iOGA proteins were distributed across 27 pathways signaling (Fig. 4f). Among the identified signaling pathways, only 8 were common to both groups, whereas 6 were unique to the control group and 19 to the iOGA group. Altogether, our data indicate that inhibition of OGA activity may disrupt the intercellular signaling via microvesicles.

**Fig. 5:**
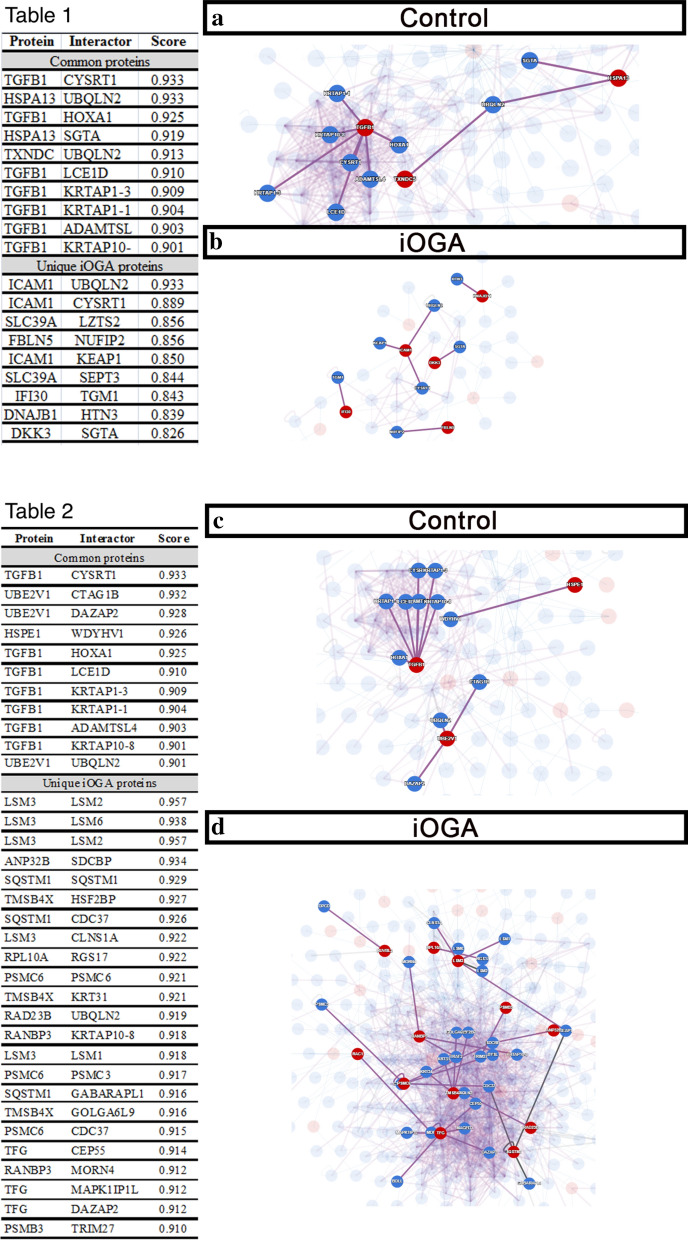
The inhibition of OGA activity alters the Interactome of GBM. Interactome analysis of proteins displaying signal peptide (Table 1) found in the control group among the 51 proteins with altered abundance (**a**) or found in the iOGA group (**b**). Interactome analysis of proteins found in GBM microvesicles (Table 2) in the control group among the 51 proteins with altered abundance (**c**) or found in the iOGA group (**d**)

Using the Human Reference Protein Interactome repository, we analyzed the putative interaction networks formed by proteins among the 51 differentially abundant proteins in iOGA that had an interaction score ≥ 0.80 [43], both among proteins that presented signal peptide and among proteins found in microvesicles. The highlighted protein abbreviations and names can be found in Additional file 8. We show that, among the 51 common proteins harboring signal peptide, the proteins TGFβ1, HSPA13 e TXNDC5 displayed high interaction score (Fig. 5a, table 1). Besides that, among the unique proteins of the iOGA group with signal peptide, the proteins ICAM1, SLC39A14, FBLN5, IFI30, DNAJB11 e DKK3 presented the strongest interactions (Fig. 5b, table 1). While in the control group we observed the prevalence of biological processes related to cell development, migration and death, in the iOGA group we observed interferon gamma signaling (IFI30) and biological processes related to the remodeling of the extra-cellular matrix, responsiveness to external stimuli and signal transduction (Fig. 5a, b).

**Fig. 6:**
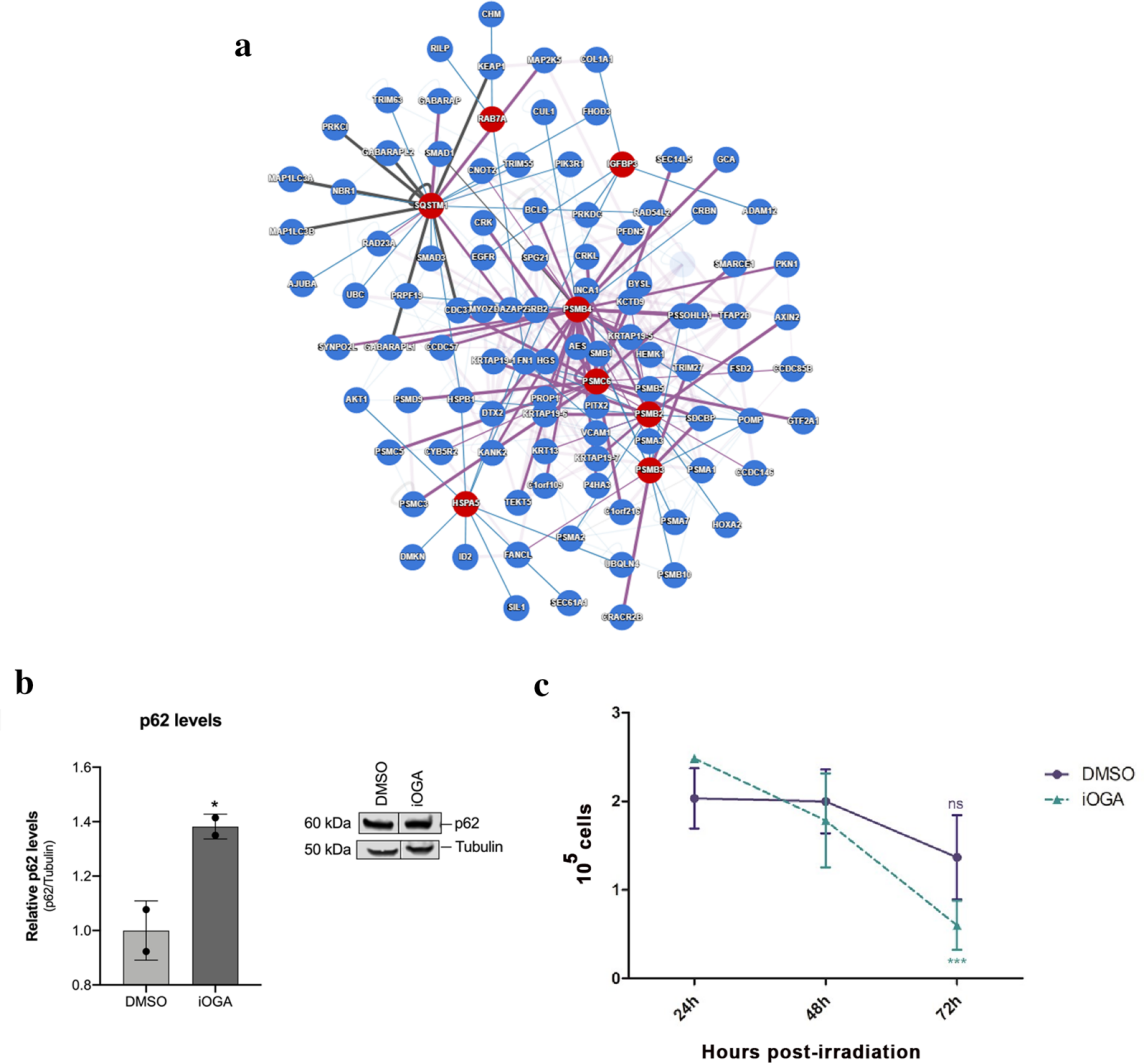
OGA inhibition increases p62 levels and reduces radioresistance in GBM. **a** Interactome of proteins related to p62 protein (IGFBP3, HSPA5/GRP78/Bip, Rab7a, SQSMT1, PSMB4, PSMB2, PSMB4 and PSMC6). **b** Western blotting and quantification analysis of p62 levels. **c** Upon 72 h of treatment control and iOGA cells were irradiated with 10 Gy and viable cells were analyzed by Neubauer count along the following 72 h post irradiation. N = 3; * *p* < 0.05; *** p < 0.001; ns = not significant

Among secretome proteins already described as being found in microvesicles, the overlapping proteins comprise TGFβ1, UBE2V1, HSPE1 (Fig. 5c, Additional file 3). As for the proteins exclusively found in the group under OGA inhibition, we highlight the presence of LSM3, ANP32B, SQSTM1, TMSB4X, RPL10A, PSMC6, RAD23B, RANBP3, TFG, RAC1, RUVBL2 and HAP1 (Fig. 5d, Additional file 3). While in the control group we observed the prevalence of TGFβ signaling (Fig. 5c), in the iOGA group we observed the formation of complexes related to proteasome and autophagy (Fig. 5d). In fact, the p62 (ubiquitin-binding protein p62, also known as SQSTM1) protein was exclusively found in the iOGA group that shows strong interaction within the signaling network (Fig. 5d). It is noteworthy to mention that the iOGA group displayed other components involved in phagosome formation, such as PSMC6 and PSMB3, which also perform strong interactions with partners within the signaling network (Fig. 5d, table 2). We also verified the presence of other unique proteins in the iOGA group that are related to endocytic pathway, such as PSMB4, PSMB2 and Rab7A (Fig. 6a; Additional file 2).

### OGA inhibition increases p62 levels and reduces radioresistance in GBM cells

Upon inhibition of OGA activity, it was possible to verify not only the emergence of p62 in the secretome, but also the levels of p62 protein were also found to be significantly elevated in cell lysates (Fig. 6a, b). Thus, our data suggests that the inhibition of OGA activity results in an increase of both accumulation and release of p62.

Since p62 has been shown to act as a non-cell tumor suppressor and decelerate tumor microenvironment aggressiveness in an autophagy-independent manner [48], we asked whether GBM cells displaying an increase in p62 abundance would contribute to mitigate the radioresistance in GBM cells. The number of cells in the control group showed a slight decay 72 h after irradiation, but without significant differences in the number of cells over time, reinforcing the concept of radioresistance of these cells (Fig. 6c). In contrast, the iOGA group showed a significant decrease in the number of viable cells over time in culture and when compared to the control group at the final time point (Fig. 6c). Thus, this data suggests that OGA inhibition plays a critical role in p62 homeostasis and consequently negatively modulates the radioresistant potential of GBM cells.

## Discussion

The identification of molecular signatures that may reflect the functional status of tumor cells might be of great value for the development of diagnostic and prognostic tools. It is well-established that GBM heterogeneity and tumor microenvironment are important for tumorigenesis with possible therapeutic implications [41]. Due to the high heterogeneity displayed by GBM cells, which is present in different GBM cell lines, we chose to use only one GBM cell line, U87-MG, that presents a mesenchymal phenotype related to high invasiveness [34]. We favored this strategy in order to generate a better interpretation of our results instead of comparing different cell lines, displaying different biological features, phenotypes and behavior, We envision that the identification of putative biomarkers can contribute to a better diagnosis and prognosis for GBM. The increase of O-GlcNAc levels is a characteristic of the majority of tumors described so far [17, 19]. For this reason, we decided to investigate the influence of increase of O-GlcNAcylation on the molecular signature of GBM secretome. For this, we induced an increase of O-GlcNAcylation by using TMG to cause inhibition of OGA activity and analyzed its impact on the U87-MG GBM cell line secretome. Interestingly, TMG has the ability to cross the blood–brain barrier, presenting potential for use in central nervous system malignancies such as GBM [22, 51].

First, we showed that TMG treatment induced a two-fold increase of O-GlcNAcylation without apparent morphological changes. In addition, we observed an increase of 25% in the number of viable cells under OGA inhibition, indicating a potential role for the O-GlcNAcylation in tumorigenesis in GBM cells. Our secretome analysis also shown that the inhibition of OGA activity disrupted the intercellular signaling via microvesicles. In fact, microvesicles and extracellular vesicles have been extensively reported as crucial for cancer progression [31, 33]. Then, we decided to characterize qualitative and quantitative differences in abundance of secretome in cells under OGA inhibition by a label-free quantitative proteomics analysis using GBM cells. We identified a total of 911 proteins, with 136 unique proteins in the control group, 103 unique proteins under OGA activity inhibition and 672 proteins common to both groups. Similarly, it has been shown that the increase in protein O-GlcNAcylation alters the adipocyte secretome during chronic insulin resistance [50]. As far as we are aware, this is the first study showing the impact of increase of O-GlcNAcylation in the secretome using a cancer cell line.

By focusing on protein whose abundance was differentially regulated upon OGA inhibition, we observed 22 upregulated proteins and 28 downregulated proteins. Among the downregulated proteins, we highlight the HSPA5 protein (heat shock 70 kDa protein 5/HSPA5/GRP78/BiP), which is an Hsp70 chaperone located in the endoplasmic reticulum involved in the folding and translocation of nascent peptide chains [13]. HSPA5 showed a 40% decrease in its abundance after treatment with TMG. This protein can directly protect cells against stress and endoplasmic reticulum damage due to reactive oxygen species in addition to playing an important role in cell survival by eliminating proteins misfolded by lysosomes through the interaction with p62, a protein uniquely found in the iOGA group [6]. Thus, HSPA5 has been proposed as an emerging therapeutic target in anti-tumor strategies [13]. In fact, high levels of HSPA5 expression have been correlated with increased malignancy and radioresistance in different tumor types [25, 47]. As for GBM, it has been shown that irradiation enhances the activation of the unfolded protein response (UPR), which is followed by increased expression of HSPA5–such phenomenon can be reversed by the administration of antibodies against HSPA5 [44]. The contributions of HSPA5 have also been shown in breast cancer, in which the HSPA5 knockdown restored the antiestrogen sensitivity in resistant tumor cells by inhibiting apoptosis and stimulating autophagy [8]. In addition, inhibition of autophagy overcame HSPA5-mediated resistance, and the simultaneous knockdown of HSPA5 and Beclin-1 restored sensitivity in resistant tumor cells [8]**.** The decrease we observed here in HSPA5 under OGA inhibition suggests that O-GlcNAcylation may play a role in reducing cell malignancy and radioresistance.

Our Volcano plot and heatmap analyses (Fig. 3) showed the downregulation of about 80% in abundance of IGFBP3 protein after OGA activity inhibition. IGFBP3 is a member of the Insulin-like Growth Factor Binding Protein (IGFBPs) family responsible for controlling the bioavailability of IGF-I and IGF-II, thus acting in the control of proliferative and anti-apoptotic signals related to these two growth factors [15]. IGFBP3 is a classic secreted protein that can interact with nuclear proteins [26, 38], being able to act on different aspects of cellular behavior such as migration, adhesion, apoptosis and proliferation [23, 40]. Chen et al. [7] demonstrated a higher expression of IGFBP3 in GBM, which was not only positively correlated with the degree of malignancy of gliomas, but also was associated with tumor histology and mutation status in isocitrate hydrogenase (IDH) 1 and 2. In addition, the higher expression of IGFBP3 was positively correlated with a worse glioma and GBM prognosis. In vitro studies suggested that IGFBP3 knockdown suppressed cell proliferation and interruption of the G2/M cell cycle, in addition to apoptosis in glioma cells [7]. Here, we further show that IGFBP3 is downregulated by OGA inhibition, suggesting once more a role of O-GlcNAcylation in reducing GBM malignancy.

It is already known the relevance of the regulation of immune response within the tumor microenvironment for the initiation and progression of the disease [24, 29]. Secretome analysis has already been proven to be a powerful tool to better understand the impact of factors released in the tumor microenvironment in the context of gliomas [2, 37]. Thus, the secretion of chemokines and cytokines by both cancer cells and stroma plays a key role in modulating this complex interaction. Within cytokines relevant for this process and also found in our secretome analysis, we highlight Interleukin-6 (IL-6)–an important cytokine produced by different solid tumors, including GBM. IL-6 has already been directly shown to be not only related to tumor progression and aggressiveness, but also found in GBM secretome [36, 45]. Also, the levels of IL-6 found in serum and cerebrospinal fluid obtained from GBM patients increases according with glioma grade and significantly decreases upon surgery [45]. IL-6 has been highlighted as a crucial component for the pro-inflammatory signature of the stroma and as a pro-tumorigenic molecule in many types of cancer [3, 14, 16]. Remarkably, our analysis revealed a downregulation of about 70% for IL-6 under OGA activity inhibition (Fig. 3, Additional file 7), adding to our findings that O-GlcNAcylation may be involved in reducing GBM progression and aggressiveness.

Interestingly, we also found that OGA inhibition was responsible for the emergence of p62 in GBM cells (Figs. 5d, 6). p62 is a protein known to aggregate with ubiquitinated proteins from the autophagic machinery allowing lysosomal degradation [4]. However, there has been increasing evidence on the role of p62 in the regulation of inflammation within the tumor microenvironment. More than that, p62 has been reported as a pro-oncogenic regulator of not only within cancer cells, but also in the modulation of the stroma [32]. During studies on p62 functions unlinked to autophagy, it has been shown that p62 loss in the stroma drives tumor progression through activation of IL-6 in cancer-associated fibroblasts [48]. These results indicate that the balance between p62 and IL-6 availability in the extracellular compartment may play a role in GBM tumor microenvironment and progression. Moreover, our data points to the relevance of p62 protein levels homeostasis in an autophagy-independent manner for GBM secretome.

Thus, our data show that OGA activity is necessary to maintain the normal composition of the GBM secretome, which in turn, may reflect in its protein repertoire the main tumor strategies in response to disturbances imposed on the tumor. The inhibition of OGA activity regulated the abundance of proteins relevant to the biology of GBM and, in particular, proteins tightly involved in regulation of inflammation in the tumor microenvironment. Our results also indicate that OGA activity is important for GBM radioresistance. This work supports studies on tumor secretome and tumor biology, which have the advantage of being easily assessed non-invasively with the use of body fluids. Certainly, the assessment of tumor status from the main proteins present in its secretome may contribute to the advancement of diagnostic, prognostic and even provide therapeutic tools to combat this relevant malignancy.

## Supplementary Information

**Additional file 1. **Sample reproducibility. **A** Principal component analysis (PCA) plot of each biological replicate and their technical replicate of control (blue) or iOGA treated cells (red). Each dot represents 3 technical replicates of 2 control biological samples (DMSO1, dark blue; DMSO2, light blue) or of 2 iOGA biological samples (TMG1, dark red; TMG2, light red). **B** VENN diagram showing unique proteins in DMSO1 (113; orange), in DMSO2 (95; dark blue) and common proteins between the two DMSO groups (808) as well as unique proteins in iOGA1 (TMG1, 47; pink), in iOGA2 (TMG2, 169; light blue) and common proteins between the two iOGA groups (775). Only common proteins to both DMSO1/2 or iOGA1/2 were used in our analysis.

**Additional file 2. **List of proteins identified with UniPotID.

**Additional file 3**. Signaling pathaway (unique proteins in iOGA group): List of unique proteins in iOGA group with UniProt ID and protin number.

**Additional file 4**. Signaling pathways (common proteins): Signaling pathways characterized among common proteins with UniProtID and pathway name.

**Additional file 5**. Signaling pathway (unique proteins in Control group): Signaling pathways characterized among unique proteins in Control group with UniProtID and pathway name.

**Additional file 6**. Comparative analysis of signaling pathways characterized in control and iOGA groups. Signaling pathways PANTHER/GO characterized in control and iOGA groups with UniProtID and indication of presence ( +) or absence (−).

**Additional file 7**. Protein fold change detected among common proteins. List of common protein and fold changes with UniProtID, protein name and signaling pathways.

**Additional file 8**. List of Highlighted Secretome Proteins.
