# Supplementary figures and images for: O-GlcNAcylation protein disruption by Thiamet G promotes changes on the GBM U87-MG cells secretome molecular signature
Source: Clin Proteomics. 2021 Apr 26;18:14. doi: 10.1186/s12014-021-09317-x (PMC8074421; doi:10.1186/s12014-021-09317-x)

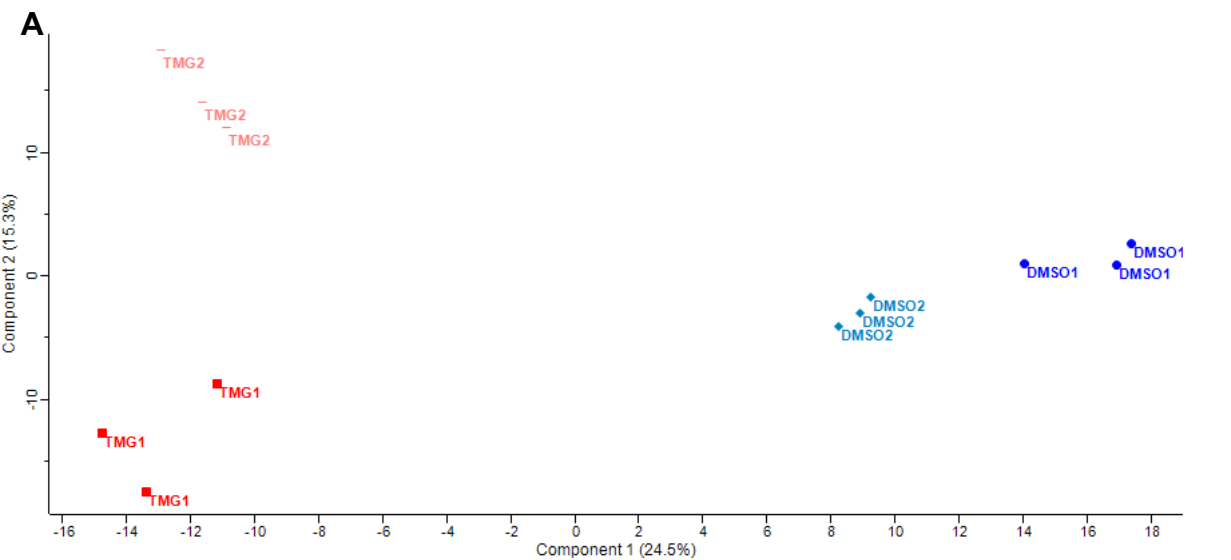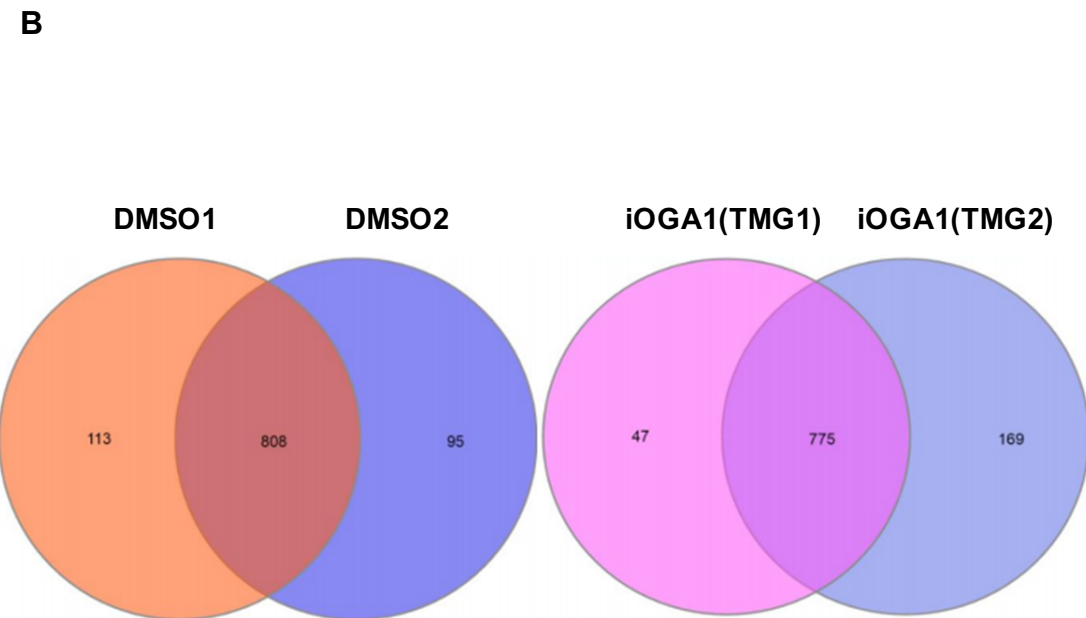

Supplement: Supplementary file 1 — Additional file 1. Sample reproducibility. A Principal component analysis (PCA) plot of each biological replicate and their technical replicate of control (blue) or iOGA treated cells (red). Each dot represents 3 technical replicates of 2 control biological samples (DMSO1, dark blue; DMSO2, light blue) or of 2 iOGA biological samples (TMG1, dark red; TMG2, light red). B VENN diagram showing unique proteins in DMSO1 (113; orange), in DMSO2 (95; dark blue) and common proteins between the two DMSO groups (808) as well as unique proteins in iOGA1 (TMG1, 47; pink), in iOGA2 (TMG2, 169; light blue) and common proteins between the two iOGA groups (775). Only common proteins to both DMSO1/2 or iOGA1/2 were used in our analysis. [file 12014_2021_9317_MOESM1_ESM.pdf]
