# Supplementary material for: O-GlcNAcylation protein disruption by Thiamet G promotes changes on the GBM U87-MG cells secretome molecular signature
Source: Clin Proteomics. 2021 Apr 26;18:14. doi: 10.1186/s12014-021-09317-x (PMC8074421; doi:10.1186/s12014-021-09317-x)
Supplement: Supplementary file 3 — Additional file 3. Signaling pathaway (unique proteins in iOGA group): List of unique proteins in iOGA group with UniProt ID and protin number. [file 12014_2021_9317_MOESM3_ESM.pdf]

Additional file 3: Signaling pathway (unique proteins in iOGA group)

UniProt	Protein name
P01903	HLA class II histocompatibility antigen, DR alpha chain (MHC class II antigen DRA)
P17301	Integrin alpha-2 (CD49 antigen-like family member B) (Collagen receptor) (Platelet membrane glycoprotein Ia) (GPIa) (VLA-2 subunit alpha) (CD antigen CD49b)
P49720	Proteasome subunit beta type-3 (EC 3.4.25.1) (Proteasome chain 13) (Proteasome component C10-II) (Proteasome theta chain)
O43396	Thioredoxin-like protein 1 (32 kDa thioredoxin-related protein)
P16989	Y-box-binding protein 3 (Cold shock domain-containing protein A) (DNA-binding protein A) (Single-strand DNA-binding protein NF-GMB)
Q14517	Protocadherin Fat 1 (Cadherin family member 7) (Cadherin-related tumor suppressor homolog) (Protein fat homolog) [Cleaved into: Protocadherin Fat 1, nuclear form]
P01008	Antithrombin-III (ATIII) (Serpin C1)
P16070	CD44 antigen (CDw44) (Epican) (Extracellular matrix receptor III) (ECMR-III) (GP90 lymphocyte homing/adhesion receptor) (HUTCH-I) (Heparan sulfate proteoglycan) (Hermes antigen) (Hyaluronate receptor) (Phagocytic glycoprotein 1) (PGP-1) (Phagocytic glycoprotein I) (PGP-I) (CD antigen CD44)
P61163	Alpha-centractin (Centractin) (ARP1) (Actin-RPV) (Centrosome-associated actin homolog)
P47895	Aldehyde dehydrogenase family 1 member A3 (EC 1.2.1.36) (Aldehyde dehydrogenase 6) (Retinaldehyde dehydrogenase 3) (RALDH-3) (RalDH3)
P11216	Glycogen phosphorylase, brain form (EC 2.4.1.1)
Q07092	Collagen alpha-1(XVI) chain
P01023	Alpha-2-macroglobulin (Alpha-2-M) (C3 and PZP-like alpha-2-macroglobulin domain-containing protein 5)
P22102	Trifunctional purine biosynthetic protein adenosine-3 [Includes: Phosphoribosylamine--glycine ligase (EC 6.3.4.13) (Glycinamide ribonucleotide synthetase) (GARS) (Phosphoribosylglycinamide synthetase); Phosphoribosylformylglycinamidine cyclo-ligase (EC 6.3.3.1) (AIR synthase) (AIRS) (Phosphoribosyl-aminoimidazole synthetase); Phosphoribosylglycinamide formyltransferase (EC 2.1.2.2) (5'-phosphoribosylglycinamide transformylase) (GAR transformylase) (GART)]
O15067	Phosphoribosylformylglycinamidine synthase (FGAM synthase) (FGAMS) (EC 6.3.5.3) (Formylglycinamide ribonucleotide amidotransferase) (FGAR amidotransferase) (FGAR-AT) (Formylglycinamide ribotide amidotransferase)
P78330	Phosphoserine phosphatase (PSP) (PSPase) (EC 3.1.3.3) (L-3-phosphoserine phosphatase) (O-phosphoserine phosphohydrolase)
P05106	Integrin beta-3 (Platelet membrane glycoprotein IIIa) (GPIIIa) (CD antigen CD61)

P40189	Interleukin-6 receptor subunit beta (IL-6 receptor subunit beta) (IL-6R subunit beta) (IL-6R-beta) (IL-6RB) (CDw130) (Interleukin-6 signal transducer) (Membrane glycoprotein 130) (gp130) (Oncostatin-M receptor subunit alpha) (CD antigen CD130)
P28070	Proteasome subunit beta type-4 (EC 3.4.25.1) (26 kDa prosomal protein) (HsBPROS26) (PROS-26) (Macropain beta chain) (Multicatalytic endopeptidase complex beta chain) (Proteasome beta chain) (Proteasome chain 3) (HsN3)
P52209	6-phosphogluconate dehydrogenase, decarboxylating (EC 1.1.1.44)
P08253	72 kDa type IV collagenase (EC 3.4.24.24) (72 kDa gelatinase) (Gelatinase A) (Matrix metalloproteinase-2) (MMP-2) (TBE-1) [Cleaved into: PEX]
Q9Y2I2	Netrin-G1 (Laminet-1)
P07737	Profilin-1 (Epididymis tissue protein Li 184a) (Profilin I)
Q9HC56	Protocadherin-9
Q03167	Transforming growth factor beta receptor type 3 (TGF-beta receptor type 3) (TGFR-3) (Betaglycan) (Transforming growth factor beta receptor III) (TGF-beta receptor type III)
Q04721	Neurogenic locus notch homolog protein 2 (Notch 2) (hN2) [Cleaved into: Notch 2 extracellular truncation (N2ECD); Notch 2 intracellular domain (N2ICD)]
P61204	ADP-ribosylation factor 3
P07814	Bifunctional glutamate/proline--tRNA ligase (Bifunctional aminoacyl-tRNA synthetase) (Cell proliferation-inducing gene 32 protein) (Glutamatyl-prolyl-tRNA synthetase) [Includes: Glutamate--tRNA ligase (EC 6.1.1.17) (Glutamyl-tRNA synthetase) (GluRS); Proline--tRNA ligase (EC 6.1.1.15) (Prolyl-tRNA synthetase)]
P05198	Eukaryotic translation initiation factor 2 subunit 1 (Eukaryotic translation initiation factor 2 subunit alpha) (eIF-2-alpha) (eIF-2A) (eIF-2alpha)
Q9BRK5	45 kDa calcium-binding protein (Cab45) (Stromal cell-derived factor 4) (SDF-4)

List of unique proteins in iOGA group with UniProt ID and protin number.
