# Supplementary material for: O-GlcNAcylation protein disruption by Thiamet G promotes changes on the GBM U87-MG cells secretome molecular signature
Source: Clin Proteomics. 2021 Apr 26;18:14. doi: 10.1186/s12014-021-09317-x (PMC8074421; doi:10.1186/s12014-021-09317-x)
Supplement: Supplementary file 4 — Additional file 4. Signaling pathways (common proteins): Signaling pathways characterized among common proteins with UniProtID and pathway name. [file 12014_2021_9317_MOESM4_ESM.pdf]

Additional file 4: Signaling pathways (common proteins):

UniProt ID	Protein name
P08758	Annexin A5 (Anchorin CII) (Annexin V) (Annexin-5) (Calphobindin I) (CBP-I) (Endonexin II) (Lipocortin V) (Placental anticoagulant protein 4) (PP4) (Placental anticoagulant protein I) (PAP-I) (Thromboplastin inhibitor) (Vascular anticoagulant-alpha) (VAC-alpha)
P50281	Matrix metalloproteinase-14 (MMP-14) (EC 3.4.24.80) (MMP-X1) (Membrane-type matrix metalloproteinase 1) (MT-MMP 1) (MTMMP1) (Membrane-type-1 matrix metalloproteinase) (MT1-MMP) (MT1MMP)
P00492	Hypoxanthine-guanine phosphoribosyltransferase (HGPRT) (HGPRTase) (EC 2.4.2.8)
Q13409	Cytoplasmic dynein 1 intermediate chain 2 (Cytoplasmic dynein intermediate chain 2) (Dynein intermediate chain 2, cytosolic) (DH IC-2)
P29120	Neuroendocrine convertase 1 (NEC 1) (EC 3.4.21.93) (Prohormone convertase 1) (Proprotein convertase 1) (PC1)
P61224	Ras-related protein Rap-1b (GTP-binding protein smg p21B)
P01137	Transforming growth factor beta-1 proprotein [Cleaved into: Latency-associated peptide (LAP); Transforming growth factor beta-1 (TGF-beta-1)]
Q99715	Collagen alpha-1(XII) chain
P00749	Urokinase-type plasminogen activator (U-plasminogen activator) (uPA) (EC 3.4.21.73) [Cleaved into: Urokinase-type plasminogen activator long chain A; Urokinase-type plasminogen activator short chain A; Urokinase-type plasminogen activator chain B]
P53396	ATP-citrate synthase (EC 2.3.3.8) (ATP-citrate (pro-S-)-lyase) (ACL) (Citrate cleavage enzyme)
P61981	14-3-3 protein gamma (Protein kinase C inhibitor protein 1) (KCIP-1) [Cleaved into: 14-3-3 protein gamma, N-terminally processed]

P32929	Cystathionine gamma-lyase (EC 4.4.1.1) (Cysteine-protein sulfhydrase) (Gamma-cystathionase)
P60900	Proteasome subunit alpha type-6 (EC 3.4.25.1) (27 kDa prosomal protein) (PROS-27) (p27K) (Macropain iota chain) (Multicatalytic endopeptidase complex iota chain) (Proteasome iota chain)
P10646	Tissue factor pathway inhibitor (TFPI) (Extrinsic pathway inhibitor) (EPI) (Lipoprotein-associated coagulation inhibitor) (LACI)
Q9BQE3	Tubulin alpha-1C chain (Alpha-tubulin 6) (Tubulin alpha-6 chain) [Cleaved into: Detyrosinated tubulin alpha-1C chain]
P14618	Pyruvate kinase PKM (EC 2.7.1.40) (Cytosolic thyroid hormone-binding protein) (CTHBP) (Opa-interacting protein 3) (OIP-3) (Pyruvate kinase 2/3) (Pyruvate kinase muscle isozyme) (Thyroid hormone-binding protein 1) (THBP1) (Tumor M2-PK) (p58)
P12109	Collagen alpha-1(VI) chain
P12111	Collagen alpha-3(VI) chain
P21589	5'-nucleotidase (5'-NT) (EC 3.1.3.5) (Ecto-5'-nucleotidase) (CD antigen CD73)
P07437	Tubulin beta chain (Tubulin beta-5 chain)
Q07954	Prolow-density lipoprotein receptor-related protein 1 (LRP-1) (Alpha-2-macroglobulin receptor) (A2MR) (Apolipoprotein E receptor) (APOER) (CD antigen CD91) [Cleaved into: Low-density lipoprotein receptor-related protein 1 85 kDa subunit (LRP-85); Low-density lipoprotein receptor-related protein 1 515 kDa subunit (LRP-515); Low-density lipoprotein receptor-related protein 1 intracellular domain (LRPICD)]
P61088	Ubiquitin-conjugating enzyme E2 N (EC 2.3.2.23) (Bendless-like ubiquitin-conjugating enzyme) (E2 ubiquitin-conjugating enzyme N) (Ubc13) (UbcH13) (Ubiquitin carrier protein N) (Ubiquitin-protein ligase N)
P10599	Thioredoxin (Trx) (ATL-derived factor) (ADF) (Surface-associated sulphydryl protein) (SASP) (allergen Hom s Trx)

Q99988	Growth/differentiation factor 15 (GDF-15) (Macrophage inhibitory cytokine 1) (MIC-1) (NSAID-activated gene 1 protein) (NAG-1) (NSAID-regulated gene 1 protein) (NRG-1) (Placental TGF-beta) (Placental bone morphogenetic protein) (Prostate differentiation factor)
P46108	Adapter molecule crk (Proto-oncogene c-Crk) (p38)
Q00610	Clathrin heavy chain 1 (Clathrin heavy chain on chromosome 17) (CLH-17)
P07996	Thrombospondin-1 (Glycoprotein G)
P54819	Adenylate kinase 2, mitochondrial (AK 2) (EC 2.7.4.3) (ATP-AMP transphosphorylase 2) (ATP:AMP phosphotransferase) (Adenylate monophosphate kinase) [Cleaved into: Adenylate kinase 2, mitochondrial, N-terminally processed]
P14324	Farnesyl pyrophosphate synthase (FPP synthase) (FPS) (EC 2.5.1.10) ((2E,6E)-farnesyl diphosphate synthase) (Dimethylallyltranstransferase) (EC 2.5.1.1) (Farnesyl diphosphate synthase) (Geranyltranstransferase)
P09429	High mobility group protein B1 (High mobility group protein 1) (HMG-1)
P13497	Bone morphogenetic protein 1 (BMP-1) (EC 3.4.24.19) (Mammalian tolloid protein) (mTld) (Procollagen C-proteinase) (PCP)
Q9BPX5	Actin-related protein 2/3 complex subunit 5-like protein (Arp2/3 complex 16 kDa subunit 2) (ARC16-2)
P16870	Carboxypeptidase E (CPE) (EC 3.4.17.10) (Carboxypeptidase H) (CPH) (Enkephalin convertase) (Prohormone-processing carboxypeptidase)
P40121	Macrophage-capping protein (Actin regulatory protein CAP-G)
O43175	D-3-phosphoglycerate dehydrogenase (3-PGDH) (EC 1.1.1.95) (2-oxoglutarate reductase) (EC 1.1.1.399) (Malate dehydrogenase) (EC 1.1.1.37)

P05067	Amyloid-beta precursor protein (APP) (ABPP) (APPI) (Alzheimer disease amyloid protein) (Amyloid precursor protein) (Amyloid-beta A4 protein) (Cerebral vascular amyloid peptide) (CVAP) (PreA4) (Protease nexin-II) (PN-II) [Cleaved into: N-APP; Soluble APP-alpha (S-APP-alpha); Soluble APP-beta (S-APP-beta); C99 (Beta-secretase C-terminal fragment) (Beta-CTF); Amyloid-beta protein 42 (Abeta42) (Beta-APP42); Amyloid-beta protein 40 (Abeta40) (Beta-APP40); C83 (Alpha-secretase C-terminal fragment) (Alpha-CTF); P3(42); P3(40); C80; Gamma-secretase C-terminal fragment 59 (Amyloid intracellular domain 59) (AICD-59) (AID(59)) (Gamma-CTF(59)); Gamma-secretase C-terminal fragment 57 (Amyloid intracellular domain 57) (AICD-57) (AID(57)) (Gamma-CTF(57)); Gamma-secretase C-terminal fragment 50 (Amyloid intracellular domain 50) (AICD-50) (AID(50)) (Gamma-CTF(50)); C31]
P28066	Proteasome subunit alpha type-5 (EC 3.4.25.1) (Macropain zeta chain) (Multicatalytic endopeptidase complex zeta chain) (Proteasome zeta chain)
O15400	Syntaxin-7
P08123	Collagen alpha-2(I) chain (Alpha-2 type I collagen)
P60174	Triosephosphate isomerase (TIM) (EC 5.3.1.1) (Methylglyoxal synthase) (EC 4.2.3.3) (Triose-phosphate isomerase)
P19883	Follistatin (FS) (Activin-binding protein)
P04632	Calpain small subunit 1 (CSS1) (Calcium-activated neutral proteinase small subunit) (CANP small subunit) (Calcium-dependent protease small subunit) (CDPS) (Calcium-dependent protease small subunit 1) (Calpain regulatory subunit)
Q9UBI6	Guanine nucleotide-binding protein G(I)/G(S)/G(O) subunit gamma-12
P39059	Collagen alpha-1(XV) chain [Cleaved into: Restin (Endostatin-XV) (Related to endostatin) (Restin-I); Restin-2 (Restin-II); Restin-3 (Restin-III); Restin-4 (Restin-IV)]

P31939	Bifunctional purine biosynthesis protein PURH [Cleaved into: Bifunctional purine biosynthesis protein PURH, N-terminally processed] [Includes: Phosphoribosylaminoimidazolecarboxamide formyltransferase (EC 2.1.2.3) (5-aminoimidazole-4-carboxamide ribonucleotide formyltransferase) (AICAR transformylase); IMP cyclohydrolase (EC 3.5.4.10) (ATIC) (IMP synthase) (Inosinicase)]
P26885	Peptidyl-prolyl cis-trans isomerase FKBP2 (PPIase FKBP2) (EC 5.2.1.8) (13 kDa FK506-binding protein) (13 kDa FKBP) (FKBP-13) (FK506-binding protein 2) (FKBP-2) (Immunophilin FKBP13) (Rotamase)
P11142	Heat shock cognate 71 kDa protein (Heat shock 70 kDa protein 8) (Lipopolysaccharide-associated protein 1) (LAP-1) (LPS-associated protein 1)
P04350	Tubulin beta-4A chain (Tubulin 5 beta) (Tubulin beta-4 chain)
P09936	Ubiquitin carboxyl-terminal hydrolase isozyme L1 (UCH-L1) (EC 3.4.19.12) (Neuron cytoplasmic protein 9.5) (PGP 9.5) (PGP9.5) (Ubiquitin thioesterase L1)
P63104	14-3-3 protein zeta/delta (Protein kinase C inhibitor protein 1) (KCIP-1)
Q13509	Tubulin beta-3 chain (Tubulin beta-4 chain) (Tubulin beta-III)
P61812	Transforming growth factor beta-2 proprotein (Cetermin) (Glioblastoma-derived T-cell suppressor factor) (G-TSF) [Cleaved into: Latency-associated peptide (LAP); Transforming growth factor beta-2 (TGF-beta-2)]
P05231	Interleukin-6 (IL-6) (B-cell stimulatory factor 2) (BSF-2) (CTL differentiation factor) (CDF) (Hybridoma growth factor) (Interferon beta-2) (IFN-beta-2)
P23528	Cofilin-1 (18 kDa phosphoprotein) (p18) (Cofilin, non-muscle isoform)
P19367	Hexokinase-1 (EC 2.7.1.1) (Brain form hexokinase) (Hexokinase type I) (HK I) (Hexokinase-A)
Q99798	Aconitate hydratase, mitochondrial (Aconitase) (EC 4.2.1.3) (Citrate hydro-lyase)
P40926	Malate dehydrogenase, mitochondrial (EC 1.1.1.37)

P07942	Laminin subunit beta-1 (Laminin B1 chain) (Laminin-1 subunit beta) (Laminin-10 subunit beta) (Laminin-12 subunit beta) (Laminin-2 subunit beta) (Laminin-6 subunit beta) (Laminin-8 subunit beta)
P16070	CD44 antigen (CDw44) (Epican) (Extracellular matrix receptor III) (ECMR-III) (GP90 lymphocyte homing/adhesion receptor) (HUTCH-I) (Heparan sulfate proteoglycan) (Hermes antigen) (Hyaluronate receptor) (Phagocytic glycoprotein 1) (PGP-1) (Phagocytic glycoprotein I) (PGP-I) (CD antigen CD44)
P20618	Proteasome subunit beta type-1 (EC 3.4.25.1) (Macropain subunit C5) (Multicatalytic endopeptidase complex subunit C5) (Proteasome component C5) (Proteasome gamma chain)
P00367	Glutamate dehydrogenase 1, mitochondrial (GDH 1) (EC 1.4.1.3)
O00764	Pyridoxal kinase (EC 2.7.1.35) (Pyridoxine kinase)
P05121	Plasminogen activator inhibitor 1 (PAI) (PAI-1) (Endothelial plasminogen activator inhibitor) (Serpin E1)
P06733	Alpha-enolase (EC 4.2.1.11) (2-phospho-D-glycerate hydro-lyase) (C-myc promoter-binding protein) (Enolase 1) (MBP-1) (MPB-1) (Non-neural enolase) (NNE) (Phosphopyruvate hydratase) (Plasminogen-binding protein)
P09972	Fructose-bisphosphate aldolase C (EC 4.1.2.13) (Brain-type aldolase)
P11021	Endoplasmic reticulum chaperone BiP (EC 3.6.4.10) (78 kDa glucose-regulated protein) (GRP-78) (Binding-immunoglobulin protein) (BiP) (Heat shock protein 70 family protein 5) (HSP70 family protein 5) (Heat shock protein family A member 5) (Immunoglobulin heavy chain-binding protein)
P30084	Enoyl-CoA hydratase, mitochondrial (EC 4.2.1.17) (Enoyl-CoA hydratase 1) (Short-chain enoyl-CoA hydratase) (SCEH)
P61158	Actin-related protein 3 (Actin-like protein 3)
P09104	Gamma-enolase (EC 4.2.1.11) (2-phospho-D-glycerate hydro-lyase) (Enolase 2) (Neural enolase) (Neuron-specific enolase) (NSE)
O15230	Laminin subunit alpha-5 (Laminin-10 subunit alpha) (Laminin-11 subunit alpha) (Laminin-15 subunit alpha)

O43707	Alpha-actinin-4 (Non-muscle alpha-actinin 4)
P07225	Vitamin K-dependent protein S
P06396	Gelsolin (AGEL) (Actin-depolymerizing factor) (ADF) (Brevin)
P62258	14-3-3 protein epsilon (14-3-3E)
P37837	Transaldolase (EC 2.2.1.2)
P28482	Mitogen-activated protein kinase 1 (MAP kinase 1) (MAPK 1) (EC 2.7.11.24) (ERT1) (Extracellular signal-regulated kinase 2) (ERK-2) (MAP kinase isoform p42) (p42-MAPK) (Mitogen-activated protein kinase 2) (MAP kinase 2) (MAPK 2)
P25789	Proteasome subunit alpha type-4 (EC 3.4.25.1) (Macropain subunit C9) (Multicatalytic endopeptidase complex subunit C9) (Proteasome component C9) (Proteasome subunit L)
P17655	Calpain-2 catalytic subunit (EC 3.4.22.53) (Calcium-activated neutral proteinase 2) (CANP 2) (Calpain M-type) (Calpain large polypeptide L2) (Calpain-2 large subunit) (Millimolar-calpain) (M-calpain)
P06744	Glucose-6-phosphate isomerase (GPI) (EC 5.3.1.9) (Autocrine motility factor) (AMF) (Neuroleukin) (NLK) (Phosphoglucose isomerase) (PGI) (Phosphohexose isomerase) (PHI) (Sperm antigen 36) (SA-36)
P21333	Filamin-A (FLN-A) (Actin-binding protein 280) (ABP-280) (Alpha-filamin) (Endothelial actin-binding protein) (Filamin-1) (Non-muscle filamin)
P27348	14-3-3 protein theta (14-3-3 protein T-cell) (14-3-3 protein tau) (Protein HS1)
P25788	Proteasome subunit alpha type-3 (EC 3.4.25.1) (Macropain subunit C8) (Multicatalytic endopeptidase complex subunit C8) (Proteasome component C8)
P68032	Actin, alpha cardiac muscle 1 (Alpha-cardiac actin) [Cleaved into: Actin, alpha cardiac muscle 1, intermediate form]
P22314	Ubiquitin-like modifier-activating enzyme 1 (EC 6.2.1.45) (Protein A1S9) (Ubiquitin-activating enzyme E1)
O14818	Proteasome subunit alpha type-7 (EC 3.4.25.1) (Proteasome subunit RC6-1) (Proteasome subunit XAPC7)

Q9UL46	Proteasome activator complex subunit 2 (11S regulator complex subunit beta) (REG-beta) (Activator of multicatalytic protease subunit 2) (Proteasome activator 28 subunit beta) (PA28b) (PA28beta)
P02452	Collagen alpha-1(I) chain (Alpha-1 type I collagen)
Q9NRA1	Platelet-derived growth factor C (PDGF-C) (Fallotein) (Spinal cord-derived growth factor) (SCDGF) (VEGF-E) [Cleaved into: Platelet-derived growth factor C, latent form (PDGFC latent form); Platelet-derived growth factor C, receptor-binding form (PDGFC receptor-binding form)]
P30086	Phosphatidylethanolamine-binding protein 1 (PEBP-1) (HCNPpp) (Neuropolypeptide h3) (Prostatic-binding protein) (Raf kinase inhibitor protein) (RKIP) [Cleaved into: Hippocampal cholinergic neurostimulating peptide (HCNP)]
Q13200	26S proteasome non-ATPase regulatory subunit 2 (26S proteasome regulatory subunit RPN1) (26S proteasome regulatory subunit S2) (26S proteasome subunit p97) (Protein 55.11) (Tumor necrosis factor type 1 receptor-associated protein 2)
O75390	Citrate synthase, mitochondrial (EC 2.3.3.1) (Citrate (Si)-synthase)
P13500	C-C motif chemokine 2 (HC11) (Monocyte chemoattractant protein 1) (Monocyte chemotactic and activating factor) (MCAF) (Monocyte chemotactic protein 1) (MCP-1) (Monocyte secretory protein JE) (Small-inducible cytokine A2)
P18206	Vinculin (Metavinculin) (MV)
P61160	Actin-related protein 2 (Actin-like protein 2)
Q03405	Urokinase plasminogen activator surface receptor (U-PAR) (uPAR) (Monocyte activation antigen Mo3) (CD antigen CD87)
Q16363	Laminin subunit alpha-4 (Laminin-14 subunit alpha) (Laminin-8 subunit alpha) (Laminin-9 subunit alpha)
P00558	Phosphoglycerate kinase 1 (EC 2.7.2.3) (Cell migration-inducing gene 10 protein) (Primer recognition protein 2) (PRP 2)

P11047	Laminin subunit gamma-1 (Laminin B2 chain) (Laminin-1 subunit gamma) (Laminin-10 subunit gamma) (Laminin-11 subunit gamma) (Laminin-2 subunit gamma) (Laminin-3 subunit gamma) (Laminin-4 subunit gamma) (Laminin-6 subunit gamma) (Laminin-7 subunit gamma) (Laminin-8 subunit gamma) (Laminin-9 subunit gamma) (S-laminin subunit gamma) (S-LAM gamma)
P14780	Matrix metalloproteinase-9 (MMP-9) (EC 3.4.24.35) (92 kDa gelatinase) (92 kDa type IV collagenase) (Gelatinase B) (GELB) [Cleaved into: 67 kDa matrix metalloproteinase-9; 82 kDa matrix metalloproteinase-9]
P35579	Myosin-9 (Cellular myosin heavy chain, type A) (Myosin heavy chain 9) (Myosin heavy chain, non-muscle IIa) (Non-muscle myosin heavy chain A) (NMMHC-A) (Non-muscle myosin heavy chain IIa) (NMMHC II-a) (NMMHC-IIA)
Q99536	Synaptic vesicle membrane protein VAT-1 homolog (EC 1.-.-.-)
Q8NES3	Beta-1,3-N-acetylglucosaminyltransferase lunatic fringe (EC 2.4.1.222) (O-fucosylpeptide 3-beta-N-acetylglucosaminyltransferase)
P99999	Cytochrome c
P19206	Biotin synthase (EC 2.8.1.6)
Q12841	Follistatin-related protein 1 (Follistatin-like protein 1)
Q16555	Dihydropyrimidinase-related protein 2 (DRP-2) (Collapsin response mediator protein 2) (CRMP-2) (N2A3) (Unc-33-like phosphoprotein 2) (ULIP-2)
O95965	Integrin beta-like protein 1 (Osteoblast-specific cysteine-rich protein) (Ten integrin EGF-like repeat domain-containing protein)
P0DP23	Calmodulin-1
P01009	Alpha-1-antitrypsin (Alpha-1 protease inhibitor) (Alpha-1-antiproteinase) (Serpin A1) [Cleaved into: Short peptide from AAT (SPAAT)]
P13521	Secretogranin-2 (Chromogranin-C) (Secretogranin II) (SgII) [Cleaved into: Secretoneurin (SN); Manserin]

P30153	Serine/threonine-protein phosphatase 2A 65 kDa regulatory subunit A alpha isoform (Medium tumor antigen-associated 61 kDa protein) (PP2A subunit A isoform PR65-alpha) (PP2A subunit A isoform R1-alpha)
Q15819	Ubiquitin-conjugating enzyme E2 variant 2 (DDVit 1) (Enterocyte differentiation-associated factor 1) (EDAF-1) (Enterocyte differentiation-promoting factor 1) (EDPF-1) (MMS2 homolog) (Vitamin D3-inducible protein)
P05556	Integrin beta-1 (Fibronectin receptor subunit beta) (Glycoprotein IIa) (GPIIA) (VLA-4 subunit beta) (CD antigen CD29)
O15144	Actin-related protein 2/3 complex subunit 2 (Arp2/3 complex 34 kDa subunit) (p34-ARC)
P46926	Glucosamine-6-phosphate isomerase 1 (EC 3.5.99.6) (Glucosamine-6-phosphate deaminase 1) (GNPDA 1) (GlcN6P deaminase 1) (Oscillin)
P52799	Ephrin-B2 (EPH-related receptor tyrosine kinase ligand 5) (LERK-5) (HTK ligand) (HTK-L)
P10600	Transforming growth factor beta-3 proprotein [Cleaved into: Latency-associated peptide (LAP); Transforming growth factor beta-3 (TGF-beta-3)]
P08648	Integrin alpha-5 (CD49 antigen-like family member E) (Fibronectin receptor subunit alpha) (Integrin alpha-F) (VLA-5) (CD antigen CD49e) [Cleaved into: Integrin alpha-5 heavy chain; Integrin alpha-5 light chain]
P49189	4-trimethylaminobutyraldehyde dehydrogenase (TMABA-DH) (TMABALDH) (EC 1.2.1.47) (Aldehyde dehydrogenase E3 isozyme) (Aldehyde dehydrogenase family 9 member A1) (EC 1.2.1.3) (Gamma-aminobutyraldehyde dehydrogenase) (EC 1.2.1.19) (R-aminobutyraldehyde dehydrogenase) [Cleaved into: 4-trimethylaminobutyraldehyde dehydrogenase, N-terminally processed]
P60709	Actin, cytoplasmic 1 (Beta-actin) [Cleaved into: Actin, cytoplasmic 1, N-terminally processed]
Q9UNN8	Endothelial protein C receptor (Activated protein C receptor) (APC receptor) (Endothelial cell protein C receptor) (CD antigen CD201)

P08833	Insulin-like growth factor-binding protein 1 (IBP-1) (IGF-binding protein 1) (IGFBP-1) (Placental protein 12) (PP12)
P09237	Matrilysin (EC 3.4.24.23) (Matrin) (Matrix metalloproteinase-7) (MMP-7) (Pump-1 protease) (Uterine metalloproteinase)
O95782	AP-2 complex subunit alpha-1 (100 kDa coated vesicle protein A) (Adaptor protein complex AP-2 subunit alpha-1) (Adaptor-related protein complex 2 subunit alpha-1) (Alpha-adaptin A) (Alpha1-adaptin) (Clathrin assembly protein complex 2 alpha-A large chain) (Plasma membrane adaptor HA2/AP2 adaptin alpha A subunit)
P04406	Glyceraldehyde-3-phosphate dehydrogenase (GAPDH) (EC 1.2.1.12) (Peptidyl-cysteine S-nitrosylase GAPDH) (EC 2.6.99.-)
Q14204	Cytoplasmic dynein 1 heavy chain 1 (Cytoplasmic dynein heavy chain 1) (Dynein heavy chain, cytosolic)
Q9H488	GDP-fucose protein O-fucosyltransferase 1 (EC 2.4.1.221) (Peptide-O-fucosyltransferase 1) (O-FucT-1)
P08254	Stromelysin-1 (SL-1) (EC 3.4.24.17) (Matrix metalloproteinase-3) (MMP-3) (Transin-1)
P09619	Platelet-derived growth factor receptor beta (PDGF-R-beta) (PDGFR-beta) (EC 2.7.10.1) (Beta platelet-derived growth factor receptor) (Beta-type platelet-derived growth factor receptor) (CD140 antigen-like family member B) (Platelet-derived growth factor receptor 1) (PDGFR-1) (CD antigen CD140b)
P19022	Cadherin-2 (CDw325) (Neural cadherin) (N-cadherin) (CD antigen CD325)
P06756	Integrin alpha-V (Vitronectin receptor) (Vitronectin receptor subunit alpha) (CD antigen CD51) [Cleaved into: Integrin alpha-V heavy chain; Integrin alpha-V light chain]
P04792	Heat shock protein beta-1 (HspB1) (28 kDa heat shock protein) (Estrogen-regulated 24 kDa protein) (Heat shock 27 kDa protein) (HSP 27) (Stress-responsive protein 27) (SRP27)

P12268	Inosine-5'-monophosphate dehydrogenase 2 (IMP dehydrogenase 2) (IMPD 2) (IMPDH 2) (EC 1.1.1.205) (IMPDH-II)
P12110	Collagen alpha-2(VI) chain
P38646	Stress-70 protein, mitochondrial (75 kDa glucose-regulated protein) (GRP-75) (Heat shock 70 kDa protein 9) (Mortalin) (MOT) (Peptide-binding protein 74) (PBP74)
O14672	Disintegrin and metalloproteinase domain-containing protein 10 (ADAM 10) (EC 3.4.24.81) (CDw156) (Kuzbanian protein homolog) (Mammalian disintegrin-metalloprotease) (CD antigen CD156c)
P31153	S-adenosylmethionine synthase isoform type-2 (AdoMet synthase 2) (EC 2.5.1.6) (Methionine adenosyltransferase 2) (MAT 2) (Methionine adenosyltransferase II) (MAT-II)
P68371	Tubulin beta-4B chain (Tubulin beta-2 chain) (Tubulin beta-2C chain)
O14786	Neuropilin-1 (Vascular endothelial cell growth factor 165 receptor) (CD antigen CD304)
P11717	Cation-independent mannose-6-phosphate receptor (CI Man-6-P receptor) (CI-MPR) (M6PR) (300 kDa mannose 6-phosphate receptor) (MPR 300) (Insulin-like growth factor 2 receptor) (Insulin-like growth factor II receptor) (IGF-II receptor) (M6P/IGF2 receptor) (M6P/IGF2R) (CD antigen CD222)
Q562R1	Beta-actin-like protein 2 (Kappa-actin)
Q9Y490	Talin-1
P55268	Laminin subunit beta-2 (Laminin B1s chain) (Laminin-11 subunit beta) (Laminin-14 subunit beta) (Laminin-15 subunit beta) (Laminin-3 subunit beta) (Laminin-4 subunit beta) (Laminin-7 subunit beta) (Laminin-9 subunit beta) (S-laminin subunit beta) (S-LAM beta)
Q9UN70	Protocadherin gamma-C3 (PCDH-gamma-C3) (Protocadherin-2) (Protocadherin-43) (PC-43)
P25787	Proteasome subunit alpha type-2 (EC 3.4.25.1) (Macropain subunit C3) (Multicatalytic endopeptidase complex subunit C3) (Proteasome component C3)

P0DMV9	Heat shock 70 kDa protein 1B (Heat shock 70 kDa protein 2) (HSP70-2) (HSP70.2)
Q9UM47	Neurogenic locus notch homolog protein 3 (Notch 3) [Cleaved into: Notch 3 extracellular truncation; Notch 3 intracellular domain]
Q04917	14-3-3 protein eta (Protein AS1)
P00491	Purine nucleoside phosphorylase (PNP) (EC 2.4.2.1) (Inosine phosphorylase) (Inosine-guanosine phosphorylase)
P11166	Solute carrier family 2, facilitated glucose transporter member 1 (Glucose transporter type 1, erythrocyte/brain) (GLUT-1) (HepG2 glucose transporter)
Q13443	Disintegrin and metalloproteinase domain-containing protein 9 (ADAM 9) (EC 3.4.24.-) (Cellular disintegrin-related protein) (Meltrin-gamma) (Metalloprotease/disintegrin/cysteine-rich protein 9) (Myeloma cell metalloproteinase)
P26006	Integrin alpha-3 (CD49 antigen-like family member C) (FRP-2) (Galactoprotein B3) (GAPB3) (VLA-3 subunit alpha) (CD antigen CD49c) [Cleaved into: Integrin alpha-3 heavy chain; Integrin alpha-3 light chain]
Q9Y617	Phosphoserine aminotransferase (EC 2.6.1.52) (Phosphohydroxythreonine aminotransferase) (PSAT)
P61769	Beta-2-microglobulin [Cleaved into: Beta-2-microglobulin form pI 5.3]
P06576	ATP synthase subunit beta, mitochondrial (EC 7.1.2.2) (ATP synthase F1 subunit beta)
P61586	Transforming protein RhoA (EC 3.6.5.2) (Rho cDNA clone 12) (h12)

Signaling pathways characterized among common proteins with UniProtID and pathway name.
