# Supplementary material for: O-GlcNAcylation protein disruption by Thiamet G promotes changes on the GBM U87-MG cells secretome molecular signature
Source: Clin Proteomics. 2021 Apr 26;18:14. doi: 10.1186/s12014-021-09317-x (PMC8074421; doi:10.1186/s12014-021-09317-x)
Supplement: Supplementary file 5 — Additional file 5. Signaling pathway (unique proteins in Control group): Signaling pathways characterized among unique proteins in Control group with UniProtID and pathway name. [file 12014_2021_9317_MOESM5_ESM.pdf]

Additonal file 5: Signaling pathway (unique proteins in Control group)

UniProt	Protein name:
Q6NZI2	Caveolae-associated protein 1 (Cavin-1) (Polymerase I and transcript release factor)
P68431	Histone H3.1 (Histone H3/a) (Histone H3/b) (Histone H3/c) (Histone H3/d) (Histone H3/f) (Histone H3/h) (Histone H3/i) (Histone H3/j) (Histone H3/k) (Histone H3/l)
P50552	Vasodilator-stimulated phosphoprotein (VASP)
O43491	Band 4.1-like protein 2 (Generally expressed protein 4.1) (4.1G)
P06132	Uroporphyrinogen decarboxylase (UPD) (URO-D) (EC 4.1.1.37)
P30520	Adenylosuccinate synthetase isozyme 2 (AMPSase 2) (AdSS 2) (EC 6.3.4.4) (Adenylosuccinate synthetase, acidic isozyme) (Adenylosuccinate synthetase, liver isozyme) (L-type adenylosuccinate synthetase) (IMP--aspartate ligase 2)
Q96AT9	Ribulose-phosphate 3-epimerase (EC 5.1.3.1) (Ribulose-5-phosphate-3-epimerase)
P17174	Aspartate aminotransferase, cytoplasmic (cAspAT) (EC 2.6.1.1) (EC 2.6.1.3) (Cysteine aminotransferase, cytoplasmic) (Cysteine transaminase, cytoplasmic) (cCAT) (Glutamate oxaloacetate transaminase 1) (Transaminase A)
P13716	Delta-aminolevulinic acid dehydratase (ALADH) (EC 4.2.1.24) (Porphobilinogen synthase)
Q9Y281	Cofilin-2 (Cofilin, muscle isoform)
P13861	cAMP-dependent protein kinase type II-alpha regulatory subunit
P30085	UMP-CMP kinase (EC 2.7.4.14) (Deoxycytidylate kinase) (CK) (dCMP kinase) (Nucleoside-diphosphate kinase) (EC 2.7.4.6) (Uridine monophosphate/cytidine monophosphate kinase) (UMP/CMP kinase) (UMP/CMPK)
Q9BYC5	Alpha-(1,6)-fucosyltransferase (Alpha1-6FucT) (EC 2.4.1.68) (Fucosyltransferase 8) (GDP-L-Fuc:N-acetyl-beta-D-glucosaminide alpha1,6-fucosyltransferase) (GDP-fucose--glycoprotein fucosyltransferase) (Glycoprotein 6-alpha-L-fucosyltransferase)

P00505	Aspartate aminotransferase, mitochondrial (mAspAT) (EC 2.6.1.1) (EC 2.6.1.7) (Fatty acid-binding protein) (FABP-1) (Glutamate oxaloacetate transaminase 2) (Kynurenine aminotransferase 4) (Kynurenine aminotransferase IV) (Kynurenine--oxoglutarate transaminase 4) (Kynurenine--oxoglutarate transaminase IV) (Plasma membrane-associated fatty acid-binding protein) (FABPpm) (Transaminase A)
Q9BUF5	Tubulin beta-6 chain (Tubulin beta class V)
P07954	Fumarate hydratase, mitochondrial (Fumarase) (HsFH) (EC 4.2.1.2)
P21399	Cytoplasmic aconitate hydratase (Aconitase) (EC 4.2.1.3) (Citrate hydro-lyase) (Ferritin repressor protein) (Iron regulatory protein 1) (IRP1) (Iron-responsive element-binding protein 1) (IRE-BP 1)
P33316	Deoxyuridine 5'-triphosphate nucleotidohydrolase, mitochondrial (dUTPase) (EC 3.6.1.23) (dUTP pyrophosphatase)

Signaling pathways characterized among unique proteins in Control group with UniProtID and pathway name.
