# Supplementary material for: O-GlcNAcylation protein disruption by Thiamet G promotes changes on the GBM U87-MG cells secretome molecular signature
Source: Clin Proteomics. 2021 Apr 26;18:14. doi: 10.1186/s12014-021-09317-x (PMC8074421; doi:10.1186/s12014-021-09317-x)
Supplement: Supplementary file 7 — Additional file 7. Protein fold change detected among common proteins. List of common protein and fold changes with UniProtID, protein name and signaling pathways. [file 12014_2021_9317_MOESM7_ESM.pdf]

	Additional file 7:	Protein fold change detected among common proteins.		
	**UniProt ID**	**Protein name:**	**Fold change**	**Signaling pathways**
**1**	O75083	WD repeat-containing protein 1 OS=Homo sapiens GN=WDR1 PE=1 SV=4	1,69133706	
**2**	P14780	Matrix metalloproteinase-9 OS=Homo sapiens GN=MMP9 PE=1 SV=3	14,73961832	1. Alzheimer disease-presenilin pathway (P00004)<br>2. CCKR signaling map (P06959)<br>3. Plasminogen activating cascade (P00050)
**3**	P55290-4	Isoform 4 of Cadherin-13 OS=Homo sapiens GN=CDH13	2,024867506	
**4**	P08254	Stromelysin-1 OS=Homo sapiens GN=MMP3 PE=1 SV=2	5,328200206	1. CCKR signaling map (P06959)<br>2. Plasminogen activating cascade (P00050)
**5**	P08195-4	Isoform 4 of 4F2 cell-surface antigen heavy chain OS=Homo sapiens GN=SLC3A2	4,897147981	
**6**	O75326	Semaphorin-7A OS=Homo sapiens GN=SEMA7A PE=1 SV=1	2,008706031	
**7**	P04156	Major prion protein OS=Homo sapiens GN=PRNP PE=1 SV=	9,196256306	
**8**	O15230	Laminin subunit alpha-5 OS=Homo sapiens GN=LAMA5 PE=1 SV=8	3,184626786	1. Integrin signalling pathway (P00034)
**9**	P04732	Metallothionein-1E OS=Homo sapiens GN=MT1E PE=1 SV=1	4,335646272	
**10**	P01137	Transforming growth factor beta-1 OS=Homo sapiens GN=TGFB1 PE=1 SV=2	1,487150806	1.TGF-beta signaling pathway (P00052)<br>2.Gonadotropin-releasing hormone

				receptor pathway (P06664)
**11**	P60900	Proteasome subunit alpha type-6 OS=Homo sapiens GN=PSMA6 PE=1 SV=1	1,456695171	1.Parkinson disease (P00049)
**12**	P17661	Desmin OS=Homo sapiens GN=DES PE=1 SV=3	5,5023044	
**13**	O15240	Neurosecretory protein VGF OS=Homo sapiens GN=VGF PE=1 SV=2	5,994563079	
**14**	P09382	Galectin-1 OS=Homo sapiens GN=LGALS1 PE=1 SV=2	1,260351848	
**15**	P06756	Integrin alpha-V OS=Homo sapiens GN=ITGAV PE=1 SV=2	2,207972661	1. CCKR signaling map (P06959)
**16**	P35442	Thrombospondin-2 OS=Homo sapiens GN=THBS2 PE=1 SV=2	1,443729215	
**17**	P54802	Alpha-N-acetylglucosaminidase OS=Homo sapiens GN=NAGLU PE=1 SV=2	2,191139298	
**18**	P13500	C-C motif chemokine 2 OS=Homo sapiens GN=CCL2 PE=1 SV=1	3,591594846	1. Inflammation mediated by chemokine and cytokine signaling pathway (P00031)
**19**	Q92859	Neogenin OS=Homo sapiens GN=NEO1 PE=1 SV=2	2,06564042	
**20**	Q13404-1	Isoform 1 of Ubiquitin-conjugating enzyme E2 variant 1 OS=Homo sapiens GN=UBE2V1	1,6856257	
**21**	P61604	10 kDa heat shock protein, mitochondrial OS=Homo sapiens GN=HSPE1 PE=1 SV=2	1,821460682	
**22**	P12268	Inosine-5'-monophosphate dehydrogenase 2 OS=Homo sapiens GN=IMPDH2 PE=1 SV=2	4,255466933	1. De novo purine biosynthesis (P02738)
**23**	Q92823-5	Isoform 5 of Neuronal cell adhesion molecule OS=Homo sapiens GN=NRCAM	0,604837186	
**24**	P61970	Nuclear transport factor 2 OS=Homo sapiens GN=NUTF2 PE=1 SV=1	0,510304087	

**25**	Q99715	Collagen alpha-1(XII) chain OS=Homo sapiens GN=COL12A1 PE=1 SV=2	0,642041426	1. Inflammation mediated by chemokine and cytokine signaling pathway (P00031)<br>2. Integrin signalling pathway (P00034)
**26**	Q9BX68	Histidine triad nucleotide-binding protein 2, mitochondrial OS=Homo sapiens GN=HINT2 PE=1 SV=1	0,376800172	
**27**	P00558	Phosphoglycerate kinase 1 OS=Homo sapiens GN=PGK1 PE=1 SV=3	0,563548047	1. Glycolysis (P00024)
**28**	Q14126	Desmoglein-2 OS=Homo sapiens GN=DSG2 PE=1 SV=2	0,480820327	
**29**	Q9BQT9-2	Isoform 2 of Calsyntenin-3 OS=Homo sapiens GN=CLSTN3	0,384473151	
**30**	P05231	Interleukin-6 OS=Homo sapiens GN=IL6 PE=1 SV=1	0,30549305	1. Inflammation mediated by chemokine and cytokine signaling pathway (P00031)<br>2. Interleukin signaling pathway (P00036)
**31**	Q9UBG0	C-type mannose receptor 2 OS=Homo sapiens GN=MRC2 PE=1 SV=2	0,483328193	
**32**	P50395	Rab GDP dissociation inhibitor beta OS=Homo sapiens GN=GDI2 PE=1 SV=2	0,78360884	
**33**	P48723	Heat shock 70 kDa protein 13 OS=Homo sapiens GN=HSPA13 PE=1 SV=1	0,365525038	
**34**	Q6UXD5	Seizure 6-like protein 2 OS=Homo sapiens GN=SEZ6L2 PE=1 SV=2	0,748990743	
**35**	O94985-2	Isoform 2 of Calsyntenin-1 OS=Homo sapiens GN=CLSTN1	0,780249101	
**36**	Q6P179	Endoplasmic reticulum aminopeptidase 2 OS=Homo sapiens GN=ERAP2 PE=1 SV=2	0,078493052	

**37**	Q04760	Lactoylglutathione lyase OS=Homo sapiens GN=GLO1 PE=1 SV=4	0,354250831	
**38**	Q8NBS9	Thioredoxin domain-containing protein 5 OS=Homo sapiens GN=TXNDC5 PE=1 SV=2	0,266357636	
**39**	P49788	Retinoic acid receptor responder protein 1 OS=Homo sapiens GN=RARRES1 PE=1 SV=2	0,143646728	
**40**	P11021	78 kDa glucose-regulated protein OS=Homo sapiens GN=HSPA5 PE=1 SV=2	0,638281563	1. Parkinson disease (P00049)<br>2. Apoptosis signaling pathway (P00006)
**41**	Q06481	Amyloid-like protein 2 OS=Homo sapiens GN=APLP2 PE=1 SV=2	0,650585032	
**42**	O60925	Prefoldin subunit 1 OS=Homo sapiens GN=PFDN1 PE=1 SV=2	0,378912773	
**43**	P51693-2	Isoform 2 of Amyloid-like protein 1 OS=Homo sapiens GN=APLP1	0,262994603	
**44**	Q14103	Heterogeneous nuclear ribonucleoprotein D0 OS=Homo sapiens GN=HNRNPD PE=1 SV=1	0,703218963	
**45**	P32119	Peroxiredoxin-2 OS=Homo sapiens GN=PRDX2 PE=1 SV=5	0,237280785	
**46**	P18669	Phosphoglycerate mutase 1 OS=Homo sapiens GN=PGAM1 PE=1 SV=2	0,31992603	
**47**	P26022	Pentraxin-related protein PTX3 OS=Homo sapiens GN=PTX3 PE=1 SV=3	0,427039135	
**48**	Q9UMF0	Intercellular adhesion molecule 5 OS=Homo sapiens GN=ICAM5 PE=1 SV=3	0,399044378	
**49**	Q9HBR0	Putative sodium-coupled neutral amino acid transporter 10 OS=Homo sapiens	0,461928235	
**50**	P01009	Alpha-1-antitrypsin OS=Homo sapiens GN=SERPINA1 PE=1 SV=3	0,392902903	1. Blood coagulation (P00011)
**51**	P17936-2	Isoform 2 of Insulin-like growth factor-binding protein 3 OS=Homo sapiens GN=IGFBP3	0,189689216	

List of common protein and fold changes with UniProtID, protein name and signaling pathways.
