# Supplementary material for: O-GlcNAcylation protein disruption by Thiamet G promotes changes on the GBM U87-MG cells secretome molecular signature
Source: Clin Proteomics. 2021 Apr 26;18:14. doi: 10.1186/s12014-021-09317-x (PMC8074421; doi:10.1186/s12014-021-09317-x)
Supplement: Supplementary file 8 — Additional file 8. List of Highlighted Secretome Proteins. [file 12014_2021_9317_MOESM8_ESM.pdf]

Additional file 8: List of Highlighted Secretome Proteins

ANP32B	acidic leucine-rich nuclear phosphoprotein 32 family member B
CCKR	cholecystokinin
CCL2	chemokine (C-C motif) ligand 2
COL12A1	collagen type XII alpha 1 chain
DKK3	dickkopf WNT signaling pathway inhibitor 3
DNAJB11	dnaJ heat shock protein family (Hsp40) member B11
EGF	epidermal growth factor
FBLN5	fibulin-5
HAP1	huntington-associated protein 1
HIF	hypoxia-induced factor
HSPA13	heat shock 70 kDa protein 13
HSPA5	heat shock 70kDa protein 5
HSPE1	heat shock protein family E (Hsp10) member 1
ICAM1	intercellular adhesion molecule 1
IFI30	gamma-interferon-inducible lysosomal thiol reductase
IGF-I	insulin-like growth factor 1
IGF-II	insulin-like growth factor 2
IGFBP3	insulin-like Growth Factor Binding Protein-3
IL6	interleukin 6
ITGAV	integrin alpha-V precursor
LSM3	U6 snRNA-associated Sm-like protein LSm3
MAPK1	mitogen-activated protein kinase 1
MMP3	metalloproteinase 3
MMP9	metalloproteinase 9
p62	ubiquitin-binding protein p62
PI3 kinase	phosphoinositide 3-kinase
PSMB2	proteasome subunit beta type-2
PSMB3	proteasome 20S subunit beta 3
PSMB4	proteasome subunit beta type-4
PSMC6	proteasome 26S Subunit, ATPase 6
Rab7A	ras-related protein Rab-7a
RAC1	ras-related C3 botulinum toxin substrate 1
RAD23B	RAD23 Homolog B, Nucleotide Excision Repair Protein
RANBP3	ran-binding protein 3
RPL10A	60S ribosomal protein L10a
RUVBL2	ruvB like AAA ATPase 2
SLC39A14	solute carrier family 39 member 14

SQSTM1	sequestosome-1
TFG	trafficking from ER to Golgi regulator
TGFβ1	transforming growth factor beta-1
TMSB4X	thymosin beta-4
TXNDC5	hioredoxin Domain Containing 5
UBE2V1	ubiquitin-conjugating enzyme E2 variant 1
VEGF	vascular endothelial growth factor
