# Supplementary material for: O-GlcNAcylation protein disruption by Thiamet G promotes changes on the GBM U87-MG cells secretome molecular signature
Source: Clin Proteomics. 2021 Apr 26;18:14. doi: 10.1186/s12014-021-09317-x (PMC8074421; doi:10.1186/s12014-021-09317-x)
Supplement: Supplementary file 8 [file 12014_2021_9317_MOESM6_ESM.doc]

	Additional file 6: Comparative analysis of signaling pathways characterized in control and iOGA groups.						
	**Signaling pathways/PANTHER/GO**	**Controle**	**iOGA**	**UniProt ID**	**Panther Family (Control)**	**UniProt ID**	**Panther family (iOGA)**
**1**	5-Hydroxytryptamine degredation (P04372)	+	+	P49189	4trimethylaminobutyraldehyde dehydrogenase	P49189<br>P47895	4trimethylaminobutyraldehyde dehydrogenase<br>Aldehyde dehydrogenase family 1 member A3
**2**	5HT1 type receptor mediated signaling pathway (P04373)	+	+	P13861<br>Q9UBI6	cAMP-dependent protein kinase type II-alpha regulatory subunit<br>Guanine nucleotide-binding protein G(I)/G(S)/G(O) subunit gamma-12	Q9UBI6	Guanine nucleotide-binding protein G(I)/G(S)/G(O) subunit gamma-12
**3**	5HT2 type receptor mediated signaling pathway (P04374)	+	+	Q9UBI6	Guaninenucleotide-biding protein G(I)/G(S)/G(0) subunit gamma-12	Q9UBI6	Guaninenucleotide-biding protein G(I)/G(S)/G(0) subunit gamma-12
**4**	5HT4 type receptor mediated signaling pathway (P04376)	+	+	Q9UBI6	Guaninenucleotide-biding protein G(I)/G(S)/G(0) subunit gamma-12	Q9UBI6	Guaninenucleotide-biding protein G(I)/G(S)/G(0) subunit gamma-12
**5**	ATP synthesis (P02721)	+	+	P99999<br>P06576	Cytochrome C<br>ATP synthase subunit beta, mitochondrial	P99999<br>P06576	Cytochrome C<br>ATP synthase subunit beta, mitochondrial
**6**	Adenine and hypoxanthine salvage pathway (P02723)	+	+	P00492<br>P00491	Hypoxanthine-guanine phosphoribosyltransferase<br>Purine nucleotide phophorylase	P00492<br>P00491	Hypoxanthine-guanine phosphoribosyltransferase<br>Purine nucleotide phophorylase
**7**	Adrenaline and noradrenaline biosynthesis (P00001)	+	+	O15400	Syntaxin-7	O15400	Syntaxin-7

**8**	Alzheimer disease-amyloid secretase pathway (P00003)	+	+	P05067<br>P29120<br>O14672<br>P28482<br>Q13443	Amyloid-beta precursor protein<br>Neuroendocrine convertase 1<br>Disintegrin and metalloproteinase domain-containing protein 10<br>Mitogen-activated protein kinase 1<br>Disintegrin and metalloproteinase domain-containing protein 9	P05067<br>P29120<br>O14672<br>Q13443	Amyloid-beta precursor protein<br>Neuroendocrine convertase 1<br>Disintegrin and metalloproteinase domain-containing protein 10<br>Disintegrin and metalloproteinase domain-containing protein 9
**9**	Alzheimer disease-presenilin pathway (P00004)	+	+	Q12841<br>P60709<br>P68032<br>P05067<br>Q562R1<br>P09237<br>P29120<br>P61160<br>Q07954<br>P16070<br>P14780<br>Q9UM47<br>P50281	Follistatin-related protein1<br>Actin, cytoplasmic 1<br>Actin, alpha cardiac muscle 1<br>Amyloid-beta precursor protein<br>Beta-actin-like-protein 2<br>Matrilysin<br>Neuroendocrine convertase 1<br>Actin-related protein 2<br>Prolow-density lipoprotein receptor-related protein 1<br>CD44 antigen	Q12841<br>P60709<br>P68032<br>P05067<br>Q562R1<br>P09237<br>P29120<br>P61160<br>Q07954<br>P16070<br>P14780<br>Q9UM47<br>P50281	Follistatin-related protein1<br>Actin, cytoplasmic 1<br>Actin, alpha cardiac muscle 1<br>Amyloid-beta precursor protein<br>Beta-actin-like-protein 2<br>Matrilysin<br>Neuroendocrine convertase 1<br>Actin-related protein 2<br>Prolow-density lipoprotein receptor-related protein 1<br>CD44 antigen<br>Matrixmetalloproteinase-

				P08253	Matrix metalloproteinase-9<br>Neurogenic locus notch homolog protein 3<br>Matrix matalloproteinase-14<br>72 kDA type IV collagenase	P08253<br>Q04721<br>P03956	9<br>Neurogenic locus notch homolog protein 3<br>Matrix matalloproteinase-14<br>72 kDA type IV collagenase<br>Neurogenic locus notch homolog protein 1<br>Interstitial collagenase
**10**	Angiogenesis (P00005)	+	+	P04792<br>P52799<br>Q9NRA1<br>P09619<br>P46108<br>P28482<br>P61586	Heat shock protein beta-1<br>Ephrin-b2<br>Platelet-derived growth factor C<br>Platelet-derived growth factor receptor beta<br>Adapter molecule crk<br>Mitogen-activated protein kinase 1<br>Transforming protein RhoA	P04792<br>P52799<br>Q9NRA1<br>P09619<br>P46108<br>P61586<br>Q04721	Heat shock protein beta-1<br>Ephrin-b2<br>Platelet-derived growth factor C<br>Platelet-derived growth factor receptor beta<br>Adapter molecule crk<br>Transforming protein RhoA<br>Neurogenic locus notch homolog protein 2
**11**	Angiotensin II-stimulated signaling through G proteins and beta-arrestin (P05911)	+	+	P01019<br>P28482<br>P61586	Angiotensinogen<br>Mitogen-activated protein kinase 1<br>Transforming protein RhoA	P01019<br>P61586	Angiotensinogen<br>Transforming protein RhoA
**12**	Apoptosis signaling pathway (P00006)	+	+	P11142<br>P0DMV9<br>P99999	Heat shock cognate 71 kDaprotein<br>Heat shock 70 kDa protein 1B<br>Cytochrome c	P11142<br>P0DMV9<br>P99999	Heat shock cognate 71 kDaprotein<br>Heat shock 70 kDa protein 1B<br>Cytochrome c

				P11021<br>P28482<br>P11717	Endoplasmic reticulum chaperone BiP<br>Mitogen-activated protein kinase1<br>Cation-independent mannose-6-phosphate receptor	P11021<br>P11717<br>P05198	Endoplasmic reticulum chaperone BiP<br>Cation-independent mannose-6-phosphate receptor<br>Eukaryotic translation initiation factor 2 subunit 1
**13**	Ascorbate degradation (P02729)	+	-	Q96AT9	Ribulose-phosphate 3-epimerase	xxxxxxxx	xxxxxxxx
**14**	Asparagine and aspartate biosynthesis (P02730)	+	+	P00505<br>P17174	Aspartate aminotransferase, mitochondrial<br>Aspartate aminotransferase, cytoplasmic		
**15**	Axon guidance mediated by Slit/Robo (P00008)	-	+	xxxxxxxx	xxxxxxxx	Q9Y2I2	Netrin-G1
**16**	Axon guidance mediated by netrin (P00009)	+	+	P50552	Vasodilator-stimulated phosphoprotein	Q9Y2I2	Netrin-G1
**17**	Axon guidance mediated by semaphorins (P00007)	+	+	O14786<br>Q16555<br>P61586	Neuropilin-1<br>Dihydropyrimidinase-related protein 2<br>Transforming protein RhoA	O14786<br>Q16555<br>P61586	Neuropilin-1<br>Dihydropyrimidinase-related protein 2<br>Transforming protein RhoA
**18**	B cell activation (P00010)	+	+	P0DP23<br>P28482	Calmodulin-1<br>Mitogen-activated protein kinase1	P0DP23<br>P28482	Calmodulin-1<br>Mitogen-activated protein kinase1

**19**	Beta1 adrenergic receptor signaling pathway (P04377)	+	+	P13861<br>Q9UBI6	cAMP-dependent protein kinase type II-alpha regulatory subunit<br>Guanine nucleotide-binding protein G(I)/G(S)/G(0) subunit gamma-12	Q9UBI6	Guanine nucleotide-binding protein G(I)/G(S)/G(0) subunit gamma-12
**20**	Beta2 adrenergic receptor signaling pathway (P04378)	+	+	P13861<br>Q9UBI6	cAMP-dependent protein kinase type II-alpha regulatory subunit<br>Guaninenucleotide-biding protein G(I)/G(S)/G(0) subunit gamma-12	Q9UBI6	Guaninenucleotide-biding protein G(I)/G(S)/G(0) subunit gamma-12
**21**	Beta3 adrenergic receptor signaling pathway (P04379)	+	+	Q9UBI6	Guaninenucleotide-biding protein G(I)/G(S)/G(0) subunit gamma-12	Q9UBI6	Guaninenucleotide-biding protein G(I)/G(S)/G(0) subunit gamma-12
**22**	Blood coagulation (P00011)	+	+	P05121<br>P07225<br>Q03405<br>P00749<br>P05067<br>P01009<br>P10646<br>Q9UNN8	Plasminogen activator inhibitor 1<br>Vitamin K-dependent protein S<br>Urokinase plasminogen activator surface receptor<br>Urokinase type plasminogen activator<br>Amyloid-beta precursor protein<br>Alpha-1-antitrypsin<br>Tissue factor pathway inhibitor<br>Endothelial proteinC receptor	P05106<br>P05121<br>P01023<br>P07225<br>Q03405<br>P00749<br>P05067<br>P01008<br>P01009<br>P10646<br>Q9UNN8	Integrin beta-3<br>Plasminogen activator inhibitor 1<br>Alpha-2-macroglobulin<br>Vitamin K-dependent protein S<br>Urokinase plasminogen activator surface receptor<br>Urokinase type plasminogen activator<br>Amyloid-beta precursor protein<br>Antithrombin-III<br>Alpha-1-antitrypsin<br>Tissue factor pathway

							inhibitor<br>Endothelial proteinC receptor
**23**	CCKR signaling map (P06959)	+	+	P04792<br>P05121<br>P00749<br>P0DP23<br>P09237<br>P08254<br>P14780<br>P46108<br>P16870<br>P28482<br>P05556<br>P61586<br>P06756	Heat shock protein beta-1<br>Plaminogen activator inhibitor 1<br>Urokinase-type plaminogen activator<br>Calmodulin-1<br>Matrilysin<br>Stromelysin-1<br>Matrix metalloproteinase-9<br>Adapter molecule crk<br>Carboxypeptidase E<br>Mitogen-activated protein kinase1<br>Integrin beta-1<br>Transforming protein RhOA<br>Integrin alpha-V	P04792<br>P05121<br>P00749<br>P0DP23<br>P09237<br>P08254<br>P14780<br>P46108<br>P16870<br>P05556<br>P61586<br>P06756	Heat shock protein beta-1<br>Plaminogen activator inhibitor 1<br>Urokinase-type plaminogen activator<br>Calmodulin-1<br>Matrilysin<br>Stromelysin-1<br>Matrix metalloproteinase-9<br>Adapter molecule crk<br>Carboxypeptidase E<br>Integrin beta-1<br>Transforming protein RhOA<br>Integrin alpha-V
**24**	Cadherin signaling pathway (P00012)	+	+	Q12841<br>P60709<br>P68032<br>P19022<br>Q562R1<br>P61160<br>Q9UN70	Follistatin-related protein 1<br>Actin, cytoplasmic 1<br>Actin, alpha cardiac muscle 1<br>Cadherin-2<br>Beta-actin-like protein 2<br>Actin-related protein 2<br>Protocadherin gamma-C3	Q12841<br>P60709<br>P68032<br>P19022<br>Q562R1<br>Q14517<br>P61160<br>Q9HC56<br>Q9UN70	Follistatin-related protein 1<br>Actin, cytoplasmic 1<br>Actin, alpha cardiac muscle 1<br>Cadherin-2<br>Beta-actin-like protein 2<br>Protocadherin Fat1<br>Actin-related protein 2<br>Protocadherin-9<br>Protocadherin gammaC3
**25**	Cell cycle (P00013)	+	+	Q9UL46	Protein activator complex	Q9UL46	Protein activator complex

					subunit 2		subunit 2
**26**	Cholesterol biosynthesis (P00014)	+	+	P14324	Farnesyl pyrophosphate synthase	P14324	Farnesyl pyrophosphate synthase
**27**	Cortocotropin releasing factor receptor signaling pathway (P04380)	+	+	Q9UBI6	Guaninenucleotide-biding protein G(I)/G(S)/G(0) subunit gamma-12	Q9UBI6	Guaninenucleotide-biding protein G(I)/G(S)/G(0) subunit gamma-12
**28**	Cytoskeletal regulation by Rho GTPase (P00016)	+	+	Q13509<br>Q9Y281<br>O15144<br>P50552<br>P60709<br>P68032<br>P07737<br>P35579<br>P23528<br>P07437<br>Q562R1<br>P68371<br>O15143<br>Q9BUF5<br>P04350<br>O15511	Tubulin beta-3 chain<br>Cofilin-2<br>Actin-related protein 2/3 complex subunit 2<br>Vasodilator-stimulated phosphoprotein<br>Actin, cytoplasmic 1<br>Actin, alpha cardiac muscle<br>Profilin-1<br>Myosin-9<br>Cofilin-1<br>Tubulin beta chain<br>Beta-actin-like protein 2<br>Tubulin beta 4B chain<br>Actin-related protein 2/3 complex subunit 1B<br>Tubulin beta-6 chain<br>Tubulin beta-4A<br>Actin-related protein 2/3 complex subunit 5	Q13509<br>O15144<br>P60709<br>P68032<br>P07737<br>P35579<br>P23528<br>P07437<br>Q562R1<br>P68371<br>Q9BUF5<br>P04350<br>O15511	Tubulin beta-3 chain<br>Actin-related protein 2/3 complex subunit 2<br>Actin, cytoplasmic 1<br>Actin, alpha cardiac muscle<br>Profilin-1<br>Myosin-9<br>Cofilin-1<br>Tubulin beta chain<br>Beta-actin-like protein 2<br>Tubulin beta-4B chain<br>Tubulin beta-6 chain<br>Tubulin beta-4A<br>Actin-related protein 2/3 complex subunit 5
**29**	DNA replication (P00017)	+	-	P68431	Histone H3.1	xxxxxxxx	xxxxxxxx

**30**	De novo purine biosynthesis (P02738)	+	+	P30520<br>P12268<br>P54819<br>P31939<br>P30085<br>P00568	Adenylosuccinate synthetase isozyme 2<br>Inosine-5’-monophosphate dehydrogenase 2<br>Adenylate kinase 2, mitochondrial<br>Bifunctional purine biosynthesis protein PURH<br>UMP-CMP kinase<br>Adenylate kinase isoenzyme 1	P22102<br>P12268<br>P54819<br>P31939<br>P00568<br>O15067	Trifunctional purine biosynthetic protein adenosine-3<br>Inosine-5’-monophosphate dehydrogenase 2<br>Adenylate kinase 2, mitochondrial<br>Bifunctional purine biosynthesis protein PURH<br>Adenylate kinase isoenzyme 1<br>Phosphoribosylformylglycimidine synthase
**31**	De novo pyrimidine deoxyribonucleotide biosynthesis (P02739)	+	-	P33316	Deoxyuridine 5’-triphosphate nucleotidohydrolase, mitochondrial	xxxxxxxx	xxxxxxxx
**32**	De novo pyrimidine ribonucleotides biosythesis (P02740)	+	-	P30085	UMP-CMP kinase	xxxxxxxx	xxxxxxxx
**33**	Dopamine receptor mediated signaling pathway (P05912)	+	+	O43491<br>P13861<br>P21333	Band 4.1-like protein 2<br>cAMP-dependent protein kinase type II-alpha regulatory subunit<br>Filamin-A	P21333	Filamin-A
**34**	EGF receptor signaling pathway (P00018)	+	+	P27348<br>P30086<br>P62258<br>Q04917<br>P61981<br>P28482<br>P63104	14-3-3 protein theta<br>Phosphatidylethanolamine-binding protein 1<br>14-3-3 protein épsilon<br>14-3-3 protein eta<br>14-3-3 protein gamma<br>Mitogen-activated protein	P27348<br>P30086<br>P62258<br>Q04917<br>P61981<br>P63104	14-3-3 protein theta<br>Phosphatidylethanolamine-binding protein 1<br>14-3-3 protein épsilon<br>14-3-3 protein eta<br>14-3-3 protein gamma<br>14-3-3 protein zeta/delta

					kinase 1<br>14-3-3 protein zeta/delta		
**35**	Endothelin signaling pathway (P00019)	+	+	P13861<br>P29120<br>P28482	cAMP-dependent protein kinase type II-alpha regulatory subunit<br>Neuroendocrine convertase 1<br>Mitogen-activated protein kinase 1	P29120	Neuroendocrine convertase 1
**36**	Enkephalin release (P05913)	+	+	P13861<br>Q9UBI6	cAMP-dependent protein kinase type II-alpha regulatory subunit<br>Guaninenucleotide-biding protein G(I)/G(S)/G(0) subunit	Q9UBI6	Guaninenucleotide-biding protein G(I)/G(S)/G(0) subunit
**37**	FAS signaling pathway (P00020)	+	+	P40121<br>P06396	Macrophage-capping protein<br>Gelsolin	P40121<br>P06396	Macrophage-capping protein<br>Gelsolin
**38**	FGF signaling pathway (P00021)	+	+	P27348<br>P30086<br>P62258<br>Q04917<br>P61981<br>P28482<br>P30153<br>P63104	14-3-3 protein theta<br>Phosphatidylethanolamine-binding protein 1<br>14-3-3 protein épsilon<br>14-3-3 protein eta<br>14-3-3 protein gamma<br>Mitogen-activated protein kinase1<br>Serine/threonine-protein phosphatase 2A 65 kDa regulatory subunit A	P27348<br>P30086<br>P62258<br>Q04917<br>P61981<br>P30153<br>P63104	14-3-3 protein theta<br>Phosphatidylethanolamine-binding protein 1<br>14-3-3 protein épsilon<br>14-3-3 protein eta<br>14-3-3 protein gamma<br>Serine/threonine-protein phosphatase 2A 65 kDa regulatory subunit A alpha isoform<br>14-3-3 protein zeta/delta

					alpha isoform<br>14-3-3 protein zeta/delta		
**39**	Fructose galactose metabolism (P02744)	+	+	P19367<br>P09972	Hexokinase-1<br>Fructose-biphosphate aldolase C	P19367<br>P09972	Hexokinase-1<br>Fructose-biphosphate aldolase C
**40**	Glutamine glutamate conversion (P02745)	-	+	xxxxxxxx	xxxxxxxx	P00367	Glutamate dehydrogenase 1, mitochondrial
**41**	GABA-B receptor II signaling (P05731)	+	-	P13861	cAMP-dependent protein kinase type II-alpha regulatory subunit	xxxxxxxx	xxxxxxxx
**42**	General transcription by RNA polymerase I (P00022)	+	-	Q6NZI2	Caveolae-associated protein 1	xxxxxxxx	xxxxxxxx
**43**	Glutamine glutamate conversion (P02745)	+	-	P00367	Glutamate dehydrogenase 1, mitochondrial	xxxxxxxx	xxxxxxxx
**44**	Glycolysis (P00024)	+	+	P04406<br>P00558<br>P14618<br>P06733<br>P06744<br>P19367<br>P60174<br>P09972<br>P09104	Glyceraldehyde-3-phosphate dehydrogenase<br>Phosphoglycerate kinase 1<br>Pyruvate kinase PKM<br>Alpha-enolase<br>Glucose-6-phosphate isomerase<br>Hexokinase-1<br>Triosephosphate isomerase<br>Fructose-biphosphate aldolaseC<br>Gamma-enolase	P04406<br>P00558<br>P14618<br>P06733<br>P06744<br>P19367<br>P60174<br>P09972<br>P09104	Glyceraldehyde-3-phosphate dehydrogenase<br>Phosphoglycerate kinase 1<br>Pyruvate kinase PKM<br>Alpha-enolase<br>Glucose-6-phosphate isomerase<br>Hexokinase-1<br>Triosephosphate isomerase<br>Fructose-biphosphate aldolaseC<br>Gamma-enolase

**45**	Gonadotropin-releasing hormone receptor pathway (P06664)	+	+	P01137<br>Q9BQE3<br>P0DMV9<br>P13521<br>P19883<br>P18206<br>P08758<br>Q9BRK5<br>P28482<br>P61224<br>P05556<br>P11166<br>P61812<br>P50281	Transforming growth factor beta-1 proprotein<br>Tubulin alpha-1C chain<br>Heat shock 70 kDA protein 1B<br>Secretogranin-2<br>Follistatin<br>Vinculin<br>Annexin A5<br>45 kDa calcium-binding protein<br>Mitogen-activated protein kinase1<br>Ras-related protein Rap-1b<br>Integrin beta-1<br>Solute carrier family 2, facilitated glucose transporter member 1<br>Transforming growth factor beta-2 proprotein<br>Matrix metalloproteinnase-14	P01137<br>Q9BQE3<br>P0DMV9<br>P13521<br>P19883<br>P18206<br>P16989<br>P08758<br>Q9BRK5<br>Q03167<br>P61224<br>P05556<br>P11166<br>P61812<br>P50281	Transforming growth factor beta-1 proprotein<br>Tubulin alpha-1C chain<br>Heat shock 70 kDA protein 1B<br>Secretogranin-2<br>Follistatin<br>Vinculin<br>Y-box-binding protein 3<br>Annexin A5<br>45 kDa calcium-binding protein<br>Transforming growth factor beta receptor type 3<br>Ras-related protein Rap-1b<br>Integrin beta-1<br>Solute carrier family 2, facilitated glucose transporter member 1<br>Transforming growth factor beta-2 proprotein<br>Matrix metalloproteinnase-14
**46**	Hedgehog signaling pathway (P00025)	+	-	P13861	cAMP-dependent protein kinase type II-alpha regulatory subunit	xxxxxxxx	xxxxxxxx
**47**	Heme biosynthesis (P02746)	+	+	P13716<br>P06132	Delta-aminolevulinic acid dehydratase<br>Uroporphynogen decarboxylase	P07814	Bifunctional glutamate/proline-tRNA ligase
**48**	Heterotrimeric G-protein signaling pathway-Gi alpha and Gs alpha mediated pathway	+	+	Q00610<br>P13861	Clathrin heavy chain 1<br>cAMP-dependent protein	Q00610<br>P0DP23	Clathrin heavy chain 1<br>Calmodulin-1

	(P00026)			P0DP23<br>Q9UBI6<br>P61224	kinase type II-alpha regulatory subunit<br>Calmodulin-1<br>Guanine nucleotide biding protein G(I)/G(S)/G(0) subunit gamma-12<br>Ras-related protein Rap-1b	P11216<br>Q9UBI6<br>P61224	Glycogen phosphorylase, brains form<br>Guanine nucleotide biding protein G(I)/G(S)/G(0) subunit gamma-12<br>Ras-related protein Rap-1b
**49**	Heterotrimeric G-protein signaling pathway-Gq alpha and Go alpha mediated pathway (P00027)	+	+	Q00610<br>Q9UBI6<br>P61224<br>P61586	Clathrin heavy chain 1<br>Guanine nucleotide biding protein G(I)/G(S)/G(0) subunit gamma-12<br>Ras-related protein Rap-1b<br>Transforming protein RhoA	Q00610<br>Q9UBI6<br>P61224<br>P61586	Clathrin heavy chain 1<br>Guanine nucleotide biding protein G(I)/G(S)/G(0) subunit gamma-12<br>Ras-related protein Rap-1b<br>Transforming protein RhoA
**50**	Heterotrimeric G-protein signaling pathway-rod outer segment phototransduction (P00028)	+	+	P0DP23<br>Q9UBI6	Calmodulin-1<br>Guanine nucleotide biding protein G(I)/G(S)/G(0) subunit gamma-12	P0DP23<br>Q9UBI6	Calmodulin-1<br>Guanine nucleotide biding protein G(I)/G(S)/G(0) subunit gamma-12
**51**	Histamine H1 receptor mediated signaling pathway (P04385)	+	+	P9UBI6	Guanine nucleotide biding protein G(I)/G(S)/G(0) subunit gamma-12	Q9UBI6	Guaninenucleotide-biding protein G(I)/G(S)/G(0) subunit
**52**	Histamine H2 receptor mediated signaling pathway (P04386)	+	+	P13861<br>Q9UBI6	cAMP-dependent protein kinase type II-alpha regulatory subunit<br>Guanine nucleotide biding protein G(I)/G(S)/G(0) subunit gamma-12	Q9UBI6	Guanine nucleotide biding protein G(I)/G(S)/G(0) subunit gamma-12
**53**	Huntington disease (P00029)	+	+	Q13509	Tubulin beta-3 chain	Q13509	Tubulin beta-3 chain

P04406	Glyceraldehyde-3-	P04406	Glyceraldehyde-3-
	phosphate dehydrogenase		phosphate dehydrogenase
P60709	Actin, cytoplasmic 1	P60709	Actin, cytoplasmic 1
P68032	Actin, alpha cardiac	P68032	Actin, alpha cardiac
	muscle 1		muscle 1
Q99536		Q99536	
Q9BPX5	Synaptic vesicle	Q9BPX5	Synaptic vesicle
	membrane protein VAT-1		membrane protein VAT-1
O95782	homolog	O95782	homolog
Q13409	Actin-related protein 2/3	Q13409	Actin-related protein 2/3
	complex subunit 5-like		complex subunit 5-like
P61204	protein	P61204	protein
P07437	AP-2 complex subunit	P07437	AP-2 complex subunit
	alpha 1		alpha 1
Q562R1		Q562R1	
P68371	Cytoplasmic dynein 1	P68371	Cytoplasmic dynein 1
	intermediate chain 2		intermediate chain 2
P61160	ADP-ribosylation factro 3	P61160	ADP-ribosylation factro 3
P17655	Tubulin beta chain	P17655	Tubulin beta chain
	Beta-actin-like protein 2		Beta-actin-like protein 2
O15143	Tubulin beta-4B chain	Q9BUF5	Tubulin beta-4B chain
Q9BUF5		Q14204	
	Actin-related protein 2		Actin-related protein 2
Q14204		P04350	
P04350	Calpain-2 catalytic	P04632	Calpain-2 catalytic
	subunit		subunit
P04632	Actin-related protein 2/3	O15511	Tubulin beta-6 chain
P61158	complex subunit 1B	P61158	Cytoplasmic dynein 1
	Tubulin beta-6 chain		heavy chain 1
			Tubulin beta-4A chain
	Cytoplasmic dynein 1		
	heavy chain 1		Calpain small subunit 1
	Tubulin beta-4A chain		Actin-related protein 2/3

					Calpain small subunit 1<br>Actin-related 2/3 complex subunit 5<br>Actin-related protein 3		complex subunit 5<br>Actin-related protein 3
**54**	Hypoxia response via HIF activation (P00030)	+	+	P10599	Thioredoxin	P10599<br>O43396	Thioredoxin<br>Thioredoxin-like protein 1
**55**	Inflammation mediated by chemokine and cytokine signaling pathway (P00031)	+	+	P05231<br>O15144<br>P60709<br>P68032<br>Q9BPX5<br>P12111<br>P35579<br>P12110<br>Q562R1<br>Q9UBI6<br>O15143<br>Q99715<br>P13500<br>P12109<br>P28482<br>O15511<br>P05556<br>P61586	Interleukin-6<br>Actin-related protein 2/3 complex subunit 2<br>Actin, cytoplasmic 1<br>Actin, alpha cardiac muscle1<br>Actin-related protein 2/3 complex subunit 5-like protein<br>Collagen alpha-3(VI) chain<br>Myosin-9<br>Collagen alpha-2(VI) chain<br>Beta-actin-like protein 2<br>Guanine nucleotide binding protein G(I)/G(S)/G(0) subunit gamma-12<br>Actin-relates protein 2/2 complex subunit 1B<br>Collagen alpha-1(XII) chain<br>C-c motif chemokine 2<br>Collagen alpha-1(VI) chain<br>Mitogen-activated protein	P05231<br>O15144<br>P17301<br>P60709<br>P68032<br>Q9BPX5<br>P12111<br>P35579<br>P12110<br>Q562R1<br>Q9UBI6<br>Q99715<br>P13500<br>P12109<br>O15511<br>P05556<br>P61586	Interleukin-6<br>Actin-related protein 2/3 complex subunit 2<br>Integrin alpha-2<br>Actin, cytoplasmic 1<br>Actin, alpha cardiac muscle 1<br>Actin-related protein 2/3 complex subunit 5-like protein<br>Collagen alpha-3(VI) chain<br>Myosin-9<br>Collagen alpha-2(VI) chain<br>Beta-actin-like protein 2<br>Guanine nucleotide binding protein G(I)/G(S)/G(0) subunit gamma-12<br>Collagen alpha-1(XII) chain<br>Collagen alpha-1(XII) chain<br>C-c motif chemokine 2<br>Actin-related protein 2/3 complex subunit 5

					kinase 1<br>Actin-related protein 2/3 complex subunit 5<br>Integrin beta-1<br>Transforming protein RhoA		Integrin beta-1<br>Transforming protein RhoA
**56**	Insulin/IGF pathway-mitogen activated protein kinase kinase/MAP kinase cascade (P00032)	+	+	P28482<br>P11717	Mitogenactivated protein kinase1<br>Cation-independent mannose-6-phosphate receptor	P11717	Cation-independent mannose-6-phosphate receptor
**57**	Insulin/IGF pathway-protein kinase B signaling cascade (P00033)	+	+	P11717	Cation-independent mannose-6- phosphate receptor	P11717	Cation-independent mannose-6- phosphate receptor
**58**	Integrin signalling pathway (P00034)	+	+	P08648<br>O15144<br>P50552<br>P60709<br>P68032<br>P07942<br>Q9BPX5<br>Q16363<br>P19206<br>P12111<br>P11047<br>P02452<br>O15230<br>P39059<br>P12110<br>Q562R1<br>P08123<br>P26006<br>P55268	Integrin alpha-5<br>Actin-related protein 2/3 complex subunit 2<br>Vasodilator-stimulated phosphoprotein<br>Actin, cytoplasmic 1<br>Actin, alpha cardiac muscle 1<br>Laminin subunit beta-1<br>Actin-related protein 2/3 complex subunit 5-like protein<br>Laminin subunit alpha-4<br>Vinculin<br>Collagen alpha-3(VI) chain<br>Laminin subunit gamma-1<br>Collagen alpha-1(I) chain<br>Laminin subunit alpha-5	P05106<br>P08648<br>O15144<br>P17301<br>P60709<br>P68032<br>P07942<br>Q9BPX5<br>Q16363<br>P19206<br>P12111<br>P11047<br>P02452<br>Q07092<br>O15230<br>P39059<br>P12110<br>Q562R1<br>P08123	Integrin beta-3<br>Actin-related protein 2/3 complex subunit 2<br>Vasodilator-stimulated phosphoprotein<br>Integrin alpha-2<br>Actin, cytoplasmic 1<br>Actin, alpha cardiac muscle 1<br>Laminin subunit beta-1<br>Actin-related protein 2/3 complex subunit 5-like protein<br>Laminin subunit alpha-4<br>Vinculin<br>Collagen alpha-3(VI) chain<br>Laminin subunit gamma-1<br>Collagen alpha-1(I) chain

				O15143<br>Q99715<br>Q9Y490<br>O43707<br>P12109<br>P46108<br>P21333<br>P28482<br>O15511<br>O95965<br>P61224	Collagen alpha-1(XV)<br>Collagen alpha-2(VI) chain<br>Beta-actin-like protein 2<br>Collagen alpha-2(I) chain<br>Integrin alpha-3<br>Laminin subunit beta-2<br>Avtin-related protein 2/3 complex subunit 1B<br>Collagen alpha-1(XII) chain<br>Talin-1<br>Alpha-actinin-4<br>Collagen alpha-1(VI) chain<br>Adapter molecule crk<br>Filamin-A<br>Mitogen-activated protein kinase 1<br>Actin-related protein 2/3 complex subunit 5<br>Integrin beta-like protein 1<br>Ras-related protein Rap-1b	P26006<br>P55268<br>Q99715<br>Q9Y490<br>O43707<br>P12109<br>P46108<br>P21333<br>O15511<br>O95965<br>P61224	Collagen alpha-1(XVI) chain<br>Laminin subunit alpha-5<br>Collagen alpha-1(XV)<br>Collagen alpha-2(VI) chain<br>Beta-actin-like protein 2<br>Collagen alpha-2(I) chain<br>Integrin alpha-3<br>Laminin subunit beta-2<br>Collagen alpha-1(XII) chain<br>Talin-1<br>Alpha-actinin-4<br>Collagen alpha-1(VI) chain<br>Adapter molecule crk<br>Filamin-A<br>Actin-related protein 2/3 complex subunit 5<br>Integrin beta-like protein 1<br>Ras-related protein Rap-1b
**59**	Interferon-gamma signaling pathway (P00035)	+	-	P28482	Mitogen-activated protein kinase 1	xxxxxxxx	xxxxxxxx
**60**	Interleukin signaling pathway (P00036)	+	+	P13861<br>Q9UBI6	cAMP-dependent protein kinase type II-alpha regulatory subunit<br>Guanine nucleotide biding protein G(I)/G(S)/G(0) subunit gamma-12	P05231<br>P40189	Interleukin-6<br>Interleukin-6 receptor subunit beta

**61**	Metabotropic glutamate receptor group II pathway (P00040)	+	+	P13861<br>Q9UBI6	cAMP-dependent protein kinase type II-alpha regulatory subunit<br>Guanine nucleotide-binding protein G(I)/G(S)/G(0) subunit gamma-12	Q9UBI6	Guanine nucleotide-binding protein G(I)/G(S)/G(0) subunit gamma-12
**62**	Metabotropic glutamate receptor group III pathway (P00039)	+	-	P13861	cAMP-dependent protein kinase type II-alpha regulatory subunit	xxxxxxxx	xxxxxxxx
**63**	Methionine biosynthesis (P02753)	+	+	P32929	Cystathionine gamma-lyase	P32929	Cystathionine gamma-lyase
**64**	Methylcitrate cycle (P02754)	+	-	P21399	Cytoplasmic aconitate hydratase	xxxxxxxx	xxxxxxxx
**65**	Muscarinic acetylcholine receptor 1 and 3 signaling pathway (P00042)	+	+	Q9UBI6	Guanine nucleotide-binding protein G(I)/G(S)/G(0) subunit gamma-12	Q9UBI6	Guanine nucleotide-binding protein G(I)/G(S)/G(0) subunit gamma-12
**66**	Muscarinic acetylcholine receptor 2 and 4 signaling pathway (P00043)	+	+	P13861<br>Q9UBI6	cAMP-dependent protein kinase type II-alpha regulatory subunit<br>Guanine nucleotide biding protein G(I)/G(S)/G(0) subunit gamma-12	Q9UBI6	Guanine nucleotide biding protein G(I)/G(S)/G(0) subunit gamma-12
**67**	N-acetylglucosamine metabolism (P02756)	+	+	P46926	Glucosamine-6-phosphate isomerase 1	P46926	Glucosamine-6-phosphate isomerase 1
**68**	Nicotine pharmacodynamics pathway (P06587)	+	+	O43491<br>P21333	Band 4.1-like protein 2<br>Filamin-A	P21333	Filamin-A
**69**	Nicotinic acetylcholine receptor signaling pathway (P00044)	+	+	P60709<br>P68032<br>P35579<br>Q562R1	Actin, cytoplasmic 1<br>Actin, alpha cardiac muscle 1<br>Myosin-9<br>Beta-actin-like protein 2	P61163<br>P60709<br>P68032<br>P35579<br>Q562R1	Alpha-centractin<br>Actin, cytoplasmic 1<br>Actin, alpha cardiac muscle 1<br>Myosin-9

							Beta-actin-like protein 2
**70**	Notch signaling pathway (P00045)	+	+	Q9BYC5<br>Q9H488<br>O14672<br>Q8NES3<br>Q9UM47	Alpha-(1,6)-fucosyltransferase<br>GDP-fucose protein O-fucosyltransferase 1<br>Disintegrin and metalloproteinase domain-containing protein 10<br>Beta-1,3-N-acetylglucosaminyltransferase lunatic fringe<br>Neurogenic locus notch homolog protein 3	Q9H488<br>O14672<br>Q8NES3<br>Q9UM47<br>Q04721	GDP-fucose protein O-fucosyltransferase 1<br>Disintegrin and metalloproteinase domain-containing protein 10<br>Beta-1,3-N-acetylglucosaminyltransferase lunatic fringe<br>Neurogenic locus notch homolog protein 3<br>Neurogenic locus notch homolog protein 2
**71**	Opioid prodynorphin pathway (P05916)	+	+	Q9UBI6	Guanine nucleotide binding protein G(I)/G(S)/G(0) subunit gamma-12	Q9UBI6	Guanine nucleotide binding protein G(I)/G(S)/G(0) subunit gamma-12
**72**	Opioid proenkephalin pathway (P05915)	+	+	Q9UBI6	Guanine nucleotide binding protein G(I)/G(S)/G(0) subunit gamma-12	Q9UBI6	Guanine nucleotide binding protein G(I)/G(S)/G(0) subunit gamma-12
**73**	Opioid proopiomelanocortin pathway (P05917)	+	+	Q9UBI6	Guanine nucleotide binding protein G(I)/G(S)/G(0) subunit gamma-12	Q9UBI6	Guanine nucleotide binding protein G(I)/G(S)/G(0) subunit gamma-12
**74**	Oxidative stress response (P00046)	+	+	P10599	Thioredoxin	P10599<br>O43396	Thioredoxin<br>Thioredoxin-like protein 1
**75**	Oxytocin receptor mediated signaling pathway (P04391)	+	+	Q9UBI6	Guanine nucleotide binding protein G(I)/G(S)/G(0) subunit gamma-12	Q9UBI6	Guanine nucleotide binding protein G(I)/G(S)/G(0) subunit gamma-12
**76**	PDGF signaling pathway (P00047)	+	+	P09619	Platelet-derived growth	P09619	Platelet-derived growth

				P28482	factor receptor beta<br>Mitogen-activated protein kinase 1		factor receptor beta
**77**	PI3 kinase pathway (P00048)	+	+	P08833<br>P63104	Insulin like growth factor-binding protein 1<br>14-3-3 protein zeta/delta	P08833<br>P63104	Insulin like growth factor-binding protein 1<br>14-3-3 protein zeta/delta
**78**	Parkinson disease (P00049)	+	+	P11142<br>P27348<br>P0DMV9<br>P25789<br>P25788<br>P20618<br>O14818<br>P62258<br>P28066<br>P60900<br>P38646<br>P25787<br>Q04917<br>P61981<br>P11021<br>O15400	Heat shock cognate 71 kDa protein<br>14-3-3 protein theta<br>Heat shock 70 kDa protein 1B<br>Proteasome subunit alpha type-4<br>Proteasome subunit alpha type-3<br>Proteasome subunit beta type-1<br>Proteasome subunit alpha type-7<br>14-3-3 protein epsilon<br>Proteasome subunit alpha type-5<br>Proteasome subunit alpha type-6<br>Stress-70 protein, mitochondrial<br>Proteasome subunit alpha	P11142<br>P27348<br>P0DMV9<br>P25789<br>P25788<br>P20618<br>O14818<br>P62258<br>P28066<br>P60900<br>P38646<br>P49720<br>P25787<br>Q04917<br>P61981<br>P11021	Heat shock cognate 71 kDa protein<br>14-3-3 protein theta<br>Heat shock 70 kDa protein 1B<br>Proteasome subunit alpha type-4<br>Proteasome subunit alpha type-3<br>Proteasome subunit beta type-1<br>Proteasome subunit alpha type-7<br>14-3-3 protein epsilon<br>Proteasome subunit alpha type-5<br>Proteasome subunit alpha type-6<br>Stress-70 protein, mitochondrial<br>Proteasoma subunit beta

				P28482<br>P09936<br>P63104	type-2<br>14-3-3 protein eta<br>Endoplasmic reticulum chaperone BiP<br>Syntaxin-7<br>Mitogen-activated protein kinase 1<br>Ubiquitin carboxyl-terminal hydrolase isozyme L1<br>14-3-3 protein zeta/delta	P28070<br>O15400<br>P09936<br>P63104	type-3<br>Proteasome subunit alpha type-2<br>14-3-3 protein eta<br>Endoplasmic reticulum chaperone BiP<br>Proteasoma subunit beta type-4<br>Syntaxin-7<br>Ubiquitin carboxyl-terminal hydrolase isozyme L1<br>14-3-3 protein zeta/delta
**79**	Pentose phosphate pathway (P02762)	+	+	P37837<br>P06744<br>P19367<br>P52209	Transaldolase<br>Glucose-6-phosphate isomerase<br>Hexokinase-1<br>6-phosphogluconate dehydrogenase, decarboxylating	P37837<br>P06744<br>P19367<br>P52209	Transaldolase<br>Glucose-6-phosphate isomerase<br>Hexokinase-1<br>6-phosphogluconate dehydrogenase, decarboxylating
**80**	Plasminogen activating cascade (P00050)	+	+	P05121<br>Q03405<br>P00749<br>P08254<br>P14780	Plasminogen activator inhibitor 1<br>Urokinase plasminogen activator surface receptor<br>Urokinase-type plasminogen activator<br>Stromelysin-1<br>Matrix metalloproteinase-9	P05121<br>Q03405<br>P00749<br>P08254<br>P14780<br>P03956	Plasminogen activator inhibitor 1<br>Urokinase plasminogen activator surface receptor<br>Urokinase-type plasminogen activator<br>Stromelysin-1<br>Matrix metalloproteinase-9<br>Intersticial collagenase
**81**	Purine metabolism (P02769)	+	+	P21589	5'-nucleotide	P21589	5'-nucleotide

				P00491	Purine nucleoside phosphorylase	P00491	Purine nucleoside phosphorylase
**82**	Pyridoxal phosphate salvage pathway (P02770)	+	+	O00764	Pyridoxal kinase	O00764	Pyridoxal kinase
**83**	Pyridoxal-5-phosphate biosynthesis (P02759)	+	+	Q9Y617	Phosphoserine aminotransferase	Q9Y617	Phosphoserine aminotransferase
**84**	Pyrimidine Metabolism (P02771)	+	+	Q16555<br>P21589	Dihydropyrimidinase-related protein 2<br>5'-nucleotidase	Q16555<br>P21589	Dihydropyrimidinase-related protein 2<br>5'-nucleotidase
**85**	Pyruvate metabolism (P02772)	+	+	P40926<br>P14618<br>P53396<br>O75390	Malate dehydrogenase, mitochondrial<br>Pyruvate kinase PKM<br>ATP-citrate synthase<br>Citrate synthase, mitochondrial	P40926<br>P14618<br>P53396<br>O75390	Malate dehydrogenase, mitochondrial<br>Pyruvate kinase PKM<br>ATP-citrate synthase<br>Citrate synthase, mitochondrial
**86**	Ras Pathway (P04393)	+	+	P28482<br>P61586	Mitogen-activated protein kinase 1<br>Transforming protein RhoA	P61586	Transforming protein RhoA
**87**	S-adenosylmethionine biosynthesis (P02773)	+	+	P31153	S-adenosylmethionene synthase isoform type-2	P31153	S-adenosylmethionene synthase isoform type-2
**88**	Salvage pyrimidine ribonucleotides (P02775)	+	**-**	P30085	UMP-CMP kinase	xxxxxxxx	xxxxxxxx
**89**	Serine glycine biosynthesis (P02776)	+	+	O43175<br>Q9Y617	D-3-phosphoglycerate dehydrogenase<br>Phosphoserine	O43175<br>Q9Y617	D-3-phosphoglycerate dehydrogenase<br>Phosphoserine

					aminotransferase	P78330	aminotransferase<br>Phosphoserine phosphatase
**90**	Succinate to proprionate conversion (P02777)	+	+	P30084	Enoyl-CoA hydratase, mitochondrial	P30084	Enoyl-CoA hydratase, mitochondrial
**91**	T cell activation (P00053)	+	+	P0DP23<br>P28482<br>P61769	Calmodulin-1<br>Mitogen-activated protein kinase 1<br>Beta-2-microglobulin	P0DP23<br>P61769<br>P01903	Calmodulin-1<br>Beta-2-microglobulin<br>HLA class II histocompatibility antigen, DR alpha chain
**92**	TCA cycle (P00051)	+	+	Q99798<br>P07954<br>O75390	Aconitate hydratase, mitochondrial<br>Fumarate hydratase, mitochondrial<br>Citrate synthase, mitochondrial	Q99798<br>O75390	Aconitate hydratase, mitochondrial<br>Citrate synthase, mitochondrial
**93**	TGF-beta signaling pathway (P00052)	+	+	P01137<br>P10600<br>P13497<br>P26885<br>P28482<br>Q99988<br>P61812	Transforming growth factor beta-1 proprotein<br>Transforming growth factor beta-3 proprotein<br>Bone morphogenetic protein 1<br>Peptidyl-prolyl cis-trans isomerase FKBP2<br>Mitogen-activated protein kinase 1<br>Growth/differentiation factor 15<br>Transforming growth	P01137<br>P10600<br>P13497<br>P26885<br>Q99988<br>P61812	Transforming growth factor beta-1 proprotein<br>Transforming growth factor beta-3 proprotein<br>Bone morphogenetic protein 1<br>Peptidyl-prolyl cis-trans isomerase FKBP2<br>Growth/differentiation factor 15<br>Transforming growth factor beta-2 proprotein

					factor beta-2 proprotein		
**94**	Thyrotropin-releasing hormone receptor signaling pathway (P04394)	+	+	Q9UBI6	Guanine nucleotide binding protein G(I)/G(S)/G(0) subunit gamma-12	Q9UBI6	Guanine nucleotide binding protein G(I)/G(S)/G(0) subunit gamma-12
**95**	Toll receptor signaling pathway (P00054)	+	+	P28482<br>P61088	Mitogen-activated protein kinase 1<br>Ubiquitin-conjugating enzyme E2N	P61088	Ubiquitin-conjugating enzyme E2N
**96**	Transcription regulation by bZIP transcription factor (P00055)	+	-	P13861	cAMP-dependent protein kinase type II-alpha regulatory subunit	xxxxxxxx	xxxxxxxx
**97**	Ubiquitin proteasome pathway (P00060)	+	+	Q13200<br>Q15819<br>P22314<br>P61088	26S proteasome non-ATPase regulatory subunit 2<br>Ubiquitin-conjugating enzyme E2 variant 2<br>Ubiquitin-like modifier-activating enzyme 1<br>Ubiquitin-conjugating enzyme E2N	Q13200<br>Q15819<br>P22314<br>P61088	26S proteasome non-ATPase regulatory subunit 2<br>Ubiquitin-conjugating enzyme E2 variant 2<br>Ubiquitin-like modifier-activating enzyme 1<br>Ubiquitin-conjugating enzyme E2N
**98**	VEGF signaling pathway (P00056)	+	+	P04792<br>P28482	Heat shock protein beta-1<br>Mitogen-activated protein kinase 1	P04792	Heat shock protein beta-1
**99**	Vasopressin synthesis (P04395)	+	+	P16870	Carboxypeptidase E	P16870	Carboxypeptidase E
**100**	Vitamin B6 metabolism (P02787)	+	+	O00764<br>Q9Y617	Pyridoxal kinase<br>Phosphoserine aminotransferase	O00764<br>Q9Y617	Pyridoxal kinase<br>Phosphoserine aminotransferase
**101**	Wnt signaling pathway (P00057)	+	+	Q12841<br>P30520	Follistatin-related protein1	Q12841<br>P60709	Follistatin-related protein1

				P60709<br>P68032<br>P19022<br>Q562R1<br>P09237<br>Q9UBI6<br>Q9UN70	Adenylosuccinate synthase isozyme 2<br>Actin, cytoplasmic 1<br>Actin, alpha cardiac muscle 1<br>Cadherin-2<br>Beta-actin-like protein 2<br>Matrilysin<br>Guanine nucleotide binding protein G(I)/G(S)/G(0) subunit gamma-12<br>Protocadherin gamma-C3	P68032<br>P19022<br>Q562R1<br>P09237<br>Q14517<br>Q9HC56<br>Q9UBI6<br>Q9UN70	Actin, cytoplasmic 1<br>Actin, alpha cardiac muscle 1<br>Cadherin-2<br>Beta-actin-like protein 2<br>Matrilysin<br>Protocadherin Fat1<br>Protocadherin-9<br>Guanine nucleotide binding protein G(I)/G(S)/G(0) subunit gamma-12<br>Protocadherin gamma-C3
**102**	Xanthine and guanine salvage pathway (P02788)	+	+	P00492<br>P00491	Hypoxanthine-guanine phosphoribosyltransferase<br>Purine nucleoside phosphorylase	P00492<br>P00491	Hypoxanthine-guanine phosphoribosyltransferase<br>Purine nucleoside phosphorylase
**103**	p38 MAPK pathway (P05918)	+	+	P04792	Heat shock protein beta-1	P04792	Heat shock protein beta-1
**104**	p53 pathway (P00059)	+	+	P05121<br>P07996<br>P09429	Plasminogen activator inhibitor 1<br>Thrombospondin-1<br>High mobility group protein B1	P05121<br>P07996<br>P09429	Plasminogen activator inhibitor 1<br>Thrombospondin-1<br>High mobility group protein B1

Signaling pathways PANTHER/GO characterized in control and iOGA groups with UniProtID and indication of presence (+) or absence (-).
